# Comparative Whole-Genome Sequence Analysis of *Alternaria alternata* KACC 411286 That Produces Alternariol-Derived Toxins and Its Secondary Metabolite Biosynthetic Potential

**DOI:** 10.3390/jof12090634

**Published:** 2026-08-24

**Authors:** Sung-Yong Hong, Ji-Su Kim, Ae-Son Om

**Affiliations:** Department of Food and Nutrition, College of Human Ecology, Hanyang University, Seoul 04763, Republic of Korea; lunohong@hanyang.ac.kr (S.-Y.H.); ssen0825@hanyang.ac.kr (J.-S.K.)

**Keywords:** alternariol-derived toxins, biosynthetic gene cluster, genome sequence, *Alternaria alternata* KACC 411286, secondary metabolites

## Abstract

*Alternaria alternata* can produce alternariol (AOH), alternariol monomethyl ether (AME), altenusin (ALN), and altenuene (ALT) on fruits and vegetables. Much is unknown about the biosynthetic gene clusters (BGCs) of secondary metabolites (SMs) including ALT in *A. alternata* isolated from strawberries. In the current study, we sequenced the whole genome of AOH- and AME-producing *A. alternata* KACC 411286 isolated from strawberry jam and carried out comparative analyses of the ALT BGC in its genome with those of other fungal strains after verification of its production of ALN and ALT. Our data showed that the assembled genome of *A. alternata* KACC 411286 is 34.2 Mb in size with 10 chromosomes. Gene Ontology analysis showed that genes involved in RNA transcription and protein synthesis and turnover are enriched in the genome of *A. alternata* KACC 411286. We identified a total of 40 SM BGCs, including the ALT BGC, in *A. alternata* KACC 411286. The comparative analysis showed that ALT BGCs are highly conserved between two *A. alternata* (KACC 411286 and ATCC 66981) and *A. arborescens* EGS 39–128. The functional conservation analyses of all six ALT biosynthetic genes also revealed that each gene in *A. alternata* KACC 411286 shares high amino acid and DNA sequence identity (above 78% identity) with its corresponding gene in four other *Alternaria* spp. except *pksI* in *A. arborescens* EGS 39–128 (55% identity at both the protein and DNA levels) and *pksI* in *A. tenuissima* BMP 0304 (53% at the DNA level). Our findings could provide a molecular basis for understanding the biosynthetic mechanisms of SMs, including ALT, in *A. alternata* KACC 411286 to reduce the contamination of fruits with multiple mycotoxins.

## 1. Introduction

The genus *Alternaria* is known to contain a diverse group of saprotrophs, plant pathogens, and opportunistic human allergens [1]. Approximately 300 *Alternaria* species have been identified worldwide, which include *Alternaria alternata*, *Alternaria arborescens*, *Alternaria tenuissima*, and *Alternaria mali* [2,3]. These *Alternaria* spp. can infect nearly 400 plant species, including economically important crops, and cause severe diseases in them, leading to economic losses. In addition, it is known that *Alternaria* spp. can produce more than 70 mycotoxins as non-host-specific and host-specific toxins [3,4]. In particular, *A. alternata* can produce non-host-specific toxins such as alternariol (AOH) and alternariol monomethyl ether (AME) on fruits and vegetables, including apples, tomatoes, and strawberries, as well as cereal grains [4,5,6]. It has been reported that AOH and AME have cytotoxic, genotoxic, mutagenic, teratogenic, and carcinogenic properties in mammalian cells [4,6,7,8,9]. Hence, recently, the European Commission (EC) has established recommended levels of AOH and AME in fruit products, including tomato products and cereal grains, including sunflower seeds (10 μg/kg of AOH and 5 μg/kg of AME in tomato products, 30 μg/kg of AOH and 30 μg/kg of AME in sunflower seeds) [10].

The biosynthetic gene cluster (BGC) of AOH-derived toxins (AME, 4-OH-AME, altenusin [ALN], altenuene [ALT]) has been reported in 5 toxin-producing *A. alternata* ATCC 66981 and *A. alternata* EOD115, which were isolated from a peanut and an apple, respectively [11,12]. It is known that the five toxins are synthesized sequentially by 4 enzymes that are encoded by the corresponding genes in the ALT BGC; the biosynthetic pathway starts with AOH synthesis by PksI, followed by OmtI to AME, MoxI to 4-OH-AME, SdrI to ALN, and finally DoxI to produce ALT [11]. However, there is no genomic study on AOH-derived toxin-producing *A. alternata* isolated from strawberries or strawberry products. Previously, we reported AOH- and AME-producing *A. alternata* KACC 411286 (formerly OM1), which was isolated from strawberry jam [13]. It is highly valuable to identify the ALT BGC in *A. alternata* KACC 411286 and to compare the organization of the BGC in this fungal strain with those in other closely related fungal species. Also, these mycotoxins are low-molecular-mass toxic secondary metabolites (SMs) and can serve not only as virulent factors to plant hosts but also as self-protective agents in unfavorable environments [14,15]. Very little information is available on SMs BGCs, including ALT BGCs in *A. alternata* isolated from strawberries or strawberry products. Thus, in this study, to gain important genomic information on SM biosynthesis in *A. alternata* KACC 411286, we sequenced the whole genome of the strain, compared the ALT BGC in the strain with those in other strains, and analyzed the BGCs of SMs, including ALT, that can be potentially produced by the strain. Our data could provide a valuable resource for understanding the biosynthetic mechanisms of SMs, including ALT, as well as AOH and AME in *Alternaria* spp. and finding effective methods to mitigate the contamination of fruits with the AOH and AME.

## 2. Materials and Methods

### 2.1. Fungal Strains and Culture Conditions

*A. alternata* KACC 411286, an AOH- and AME-producing strain isolated from strawberry jam as described previously [13], was used in this study.

To investigate the effects of strawberry extracts on AOH and AME production by *A. alternata* KACC 411286 and its production of other AOH-derived toxins, approximately 10^6^ fungal spores were inoculated into 5 mL of potato dextrose broth (PDB; MB Cell, Seoul, Republic of Korea) or PDB supplemented with strawberry extracts (cultivar Sulhyang), for which the extract was added to PDB at a 10% (*v*/*v*) concentration after being homogenized and filtered using 0.2 μm polyvinylidene fluoride (PVDF) syringe filter (Hyundai Micro Co., Seoul, Republic of Korea), and cultured at 25 °C for 5 days under static conditions.

To analyze the relative expression of AOH-derived toxin biosynthetic genes in the cluster by RT-qPCR, 10^7^ fungal spores were inoculated into 100 mL of yeast extract sucrose (YES; 2% yeast extract, 15% sucrose, 0.1% MgSO_4_·H_2_O) or malt extract broth (MEB; MB Cell, Seoul, Republic of Korea) liquid medium and incubated at 25 °C for 10 days under static (for YES) or agitation conditions (for MEB) at 150 rpm.

To isolate genomic DNA, approximately 10^7^ fungal spores were inoculated into 100 mL of YES and incubated at 25 °C for 8 days without shaking. For total RNA isolation, the fungal strain was cultured in 100 mL of YES under static conditions and 100 mL of MEB under agitation conditions at 25 °C for 8 days.

### 2.2. Preparation of AOH and AME Standard Solutions and Extraction of the Toxins from Fungal Cultures

An AOH or AME stock solution (200 μg/mL) was made by dissolving 5 mg of AOH or AME powder (Sigma-Aldrich Co., St. Louis, MO, USA) in 25 mL of methanol (MeOH). Then, a series of AOH or AME standards were prepared freshly by dilutions of the stock solution with MeOH at concentrations of 0.1, 0.2, 0.5, 1.0, and 2.0 μg/mL.

AOH, AME, and AOH-derived toxin extractions were performed by the method described by Patriaca and collaborators with some modifications as described previously [13,16]. 

### 2.3. HPLC Analysis

An HPLC system (LC-20AT, Shimadzu; Tokyo, Japan) equipped with a UV detector (UVD; SPD-10A, Shimadzu; Tokyo, Japan) was used to detect and quantify AOH and AME at 258 nm. The toxins were separated on a ZORBAX Eclips plus C18 column (4.6 mm × 250 mm, 5 μm particle size, Agilent; Santa Clara, CA, USA) at a flow rate of 0.4 mL/min, and the mobile phase was 80% MeOH (*v*/*v*) containing 300 mg of zinc sulfate (ZnSO_4_·H_2_O)/L as described previously [13]. The column oven temperature was set at 30 °C and the injection volume of samples was 10 μL.

The linearity of a series of AOH and AME concentrations in the HPLC-UVD analysis was evaluated by a standard curve using standard solutions of each type of toxin described above. The linearity was analyzed by linear regression and expressed as a coefficient of determination (r^2^). Both AOH and AME standard curves showed an r^2^ value of 0.999 (Figure S1).

The sensitivity of the analytical method using HPLC-UVD was determined by the limit of quantification (LOQ) and limit of detection (LOD). They were calculated using the slope (S) of the calibration curve and the standard deviation (SD) of the response as follows:

$$LOQ=\frac{10\times SD}{\begin{array}{c} S \end{array}}$$

$$LOD=\frac{3.3\times SD}{\begin{array}{c} S \end{array}}$$

The LOQ and LOD for AOH were 0.06 and 0.02 μg/mL, whereas those for AME were 0.017 and 0.005 μg/mL, respectively.

### 2.4. LC/Q-TOF Analysis

Liquid chromatography/Quadrupole Time-of-Flight (LC/Q-TOF) was used to check whether *A. alternata* KACC 411286 produces other AOH-derived toxins as well as AOH and AME. The LC/Q-TOF analysis was performed using an Agilent 1290 Infinity UHPLC system (Agilent, Santa Clara, CA, USA), which was coupled to an Agilent 6545XT Q-TOF mass (MS) spectrometer (Agilent, Santa Clara, CA, USA) equipped with a dual-spray Agilent Jet Stream (AJS) electrospray ionization (ESI) source. Separation was carried out on an Agilent ZORBAX Eclipse Plus C18 column (150 mm × 2.1 mm, 3.5 μm particle size; Santa Clara, CA, USA) through an on-line mixture of solvent A (100% pure water) and solvent B (100% acetonitrile [ACN]) as a mobile phase with a flow rate of 0.3 mL/min. The column oven temperature was maintained at 30 °C and the injection volume of samples was 20 μL. The applied gradient elution program was as follows: after solution B linearly increased to 5% from 0 min to 2 min, it continuously increased to 95% from 2 min to 29 min. Then, it was held at 95% from 29 min to 30 min. Subsequently, the 95% B solution rapidly decreased from 95% at 30 min to 5% at 31 min.

The MS spectrometer was operated in the positive and negative modes as follows: capillary voltage, 3500 V; nozzle voltage, 300 V; drying gas temperature, 325 °C; drying gas flow rate, 6 L/min; sheath gas temperature, 350 °C; sheath gas flow rate, 11 L/min; nebulizer gas pressure, 45 psi. The peak spectrum was acquired using the Find by Formula data-mining algorithm. Agilent MassHunter Qualitative Analysis Software (rev. 10.0; Santa Clara, CA, USA) was used for data processing.

The same LC/Q-TOF system was used to identify SMs produced by *A. alternata* KACC 411286 based on tandem mass spectrometry (MS/MS). Separation was carried out on an Agilent ZORBAX Eclipse Plus C18 column (100 mm × 2.1 mm, 1.8 μm particle size; Agilent, Santa Clara, CA, USA) using solvent A (100% pure water) and solvent B (100% ACN) as a mobile phase with a flow rate of 0.3 mL/min. The column oven temperature was maintained at 35 °C and the injection volume of samples was 5 μL. The detailed gradient elution program was as follows: after B solution linearly increased to 10% from 0 min to 2 min, it continuously increased to 90% from 2 min to 10 min. Then, it was maintained at 90% from 10 min to 12 min. Subsequently, the 90% solution B rapidly decreased from 90% at 12 min to 10% at 17 min.

The MS spectrometer was operated in the negative mode using the same parameters as described above, except for a collision energy of 20 eV.

### 2.5. Relative Gene Expression Analysis by RT-qPCR

After *A. alternata* KACC 411286 was cultured in YES without shaking or MEB with shaking and harvested as described above, the mycelia were promptly frozen in liquid N_2_ for total RNA extraction. Total RNA was extracted from the mycelia using the RNeasy Mini Kit (QIAGEN, Hilden, Germany) according to the manufacturer’s instructions. The RNA quality was examined using an Agilent 2100 Bioanalyzer (Agilent Technologies, Santa Clara, CA, USA).

Specific primer sets for the analysis of AOH-derived toxin biosynthetic genes were designed using Primer3Plus on the basis of the genome sequence of *A. alternata* KACC 411286 (Table S1) [17,18]. The specificity of the primer sequences was evaluated using the Primer-BLAST at the National Center for Biotechnology Information (NCBI) website [19]. The β-tubulin gene of *A. alternata* NSH–1 (GenBank accession number MN175551) was used to design a specific set of primers for normalization [20].

cDNA synthesis was carried out using the PrimerScript RT Reagent Kit with gDNA Eraser (Perfect Real Time) from TaKaRa (Takara Bio Inc., Shiga, Japan) following a protocol provided by the manufacturer, as described previously [21]. The TB Green Premix Ex Taq II (Tli RNaseH Plus) ROX plus Kit (Takara Bio Inc., Shiga, Japan) was used for the RT-qPCR reaction following a procedure provided by the manufacturer, as described previously [21]. RT-qPCR reactions were conducted under the same conditions as described previously [13]. The data were analyzed by the relative quantification method using CFX Maestro software (v. 4.1; Bio-Rad, Hercules, CA, USA), and the relative gene expression level was calculated by the 2^−△△^CT method [22]. The RT-qPCR analysis was performed with three independent biological replicates, and two replicates were analyzed for each biological sample.

### 2.6. Statistical Analyses

Statistical analyses were performed using a one-way analysis of variance (ANOVA), which was followed by Duncan’s multiple-range test as a post hoc analysis. Data were represented as the mean ± SD using SPSS 26.0 (SPSS Inc., Chicago, IL, USA). A *p* value < 0.05 was considered statistically significantly different between data.

### 2.7. Genomic DNA Extraction for Whole Genome Sequencing and Total RNA Isolation for RNA-Seq

To isolate genomic DNA from *A. alternata* KACC 411286, after fungal culture in YES as described above, mycelia were filtered through miracloth (Sigma-Aldrich, Burlington, MA, USA) and rapidly frozen in liquid N_2_. The frozen mycelia were used to isolate genomic DNA using a modified CTAB method with 2% polyvinylpyrrolidone (PVP; 1% with MW 10,000 and 1% with MW 40,000) (Sigma-Aldrich Co., St. Louis, MO, USA) [23] at eGnome Co., Ltd. (Seoul, Republic of Korea) as described previously [21]. The isolated genomic DNA was then stored at −20 °C until further use.

To isolate total RNA from *A. alternata* KACC 411286, after fungal culture in YES and MEB media and filtration of mycelia as described above, the mycelia were ground into a fine powder under liquid N_2_ using a mortar and pestle. Total RNA was then extracted using the QIAGEN RNeasy Mini Kit (QIAGEN, Hilden, Germany) according to the manufacturer’s instructions, as described previously [21].

### 2.8. Genome Sequencing and Assembly

The genome of *A. alternata* KACC 411286 was sequenced using MinION Mk1B (Oxford Nanopore Technologies, Oxford, UK) at eGnome Co., Ltd. (Seoul, Republic of Korea) as described previously [21]. Short DNA fragments (<10 kb) were removed using the Short Read Eliminator Kit (Circuomics, Baltimore, MD, USA) to selectively sequence long fragments. Then, the sequencing library was constructed using the ONT Ligation Sequencing Kit V14 (SQK-LSK114; Oxford Nanopore Technologies) following the instructions provided by the manufacturer. The DNA library was loaded onto a MinION flow cell (FLO-MIN114, R10.4.1; Oxford Nanopore Technologies), and sequencing was carried out on a MinION Mk1B platform using MinKNOW software (v24.11.4). In total, 369,871 reads with an average length of 81,667.7 base pairs (bp) were obtained as raw sequencing data.

After basecalling with Dorado (v7.4.14), raw reads were initially processed to remove adapters using Porechop (v0.2.4) [24]. Low-quality reads (quality score < 14, read length < 2000 bp) were further removed from the sequencing data using NanoFilt (v2.8.0) [25]. The filtered data produced 223,261 reads with an average length of 11,607.0 bp. The quality of both raw and filtered sequencing data was evaluated using NanoPlot (v1.41.0) [26]. The sequences were assembled into a draft genome using Flye (v2.9.3-b1797) with the following option ‘–nano-hq mode, –asm_coverage 50, and –genome-size 33M’ [27]. The quality of the assembled genome was assessed using the Quality Assessment Tool for Genome Assemblies (QUAST; v5.1.0rc1) [28]. To further evaluate the completeness of the genome assembly, Benchmarking Universal Single-Copy Orthologs (BUSCO; v5.4.4) scores were analyzed against the ‘pleosporales_odb10’ database under the genome mode [29]. Due to its high BUSCO scores (99.22%), the draft genome was deemed to be of high quality and used as the final assembled genome.

### 2.9. RNA-Seq Analysis

The RNA-sequencing (RNA-Seq) analysis for *A. alternata* KACC 411286 was carried out using Illumina NovaSeq 6000 (Illumina Inc., San Diego, CA, USA) at eGnome Co., Ltd. (Seoul, Republic of Korea). Briefly, after mRNA isolation and cDNA synthesis, an RNA-Seq library was constructed with cDNA fragments using the NEBNext Ultra Directional RNA Library Prep Kit for Illumina (New England Biolabs, Ipswich, MA, USA) following the procedure provided by the manufacturer, as described previously [21]. The RNA-Seq library was assessed for size distribution and quality of the cDNA fragments using an Agilent 4200 TapeStation system and sequenced on Illumina NovaSeq 6000 (Illumina Inc.) with the options ‘100 bp paired-end reads’. The quality of the Illumina RNA-Seq data was further evaluated using FastQC (v0.12.1; https://github.com/s-andrews/FastQC/). The quality control checks on Illumina RNA-Seq data using FastQC produced a total of 27,292,587 and 27,339,585 read-pairs from YES and MEB cultures of the strain, respectively. The paired-end reads from RNA-Seq libraries were trimmed to remove adapter sequences using Trimmomatic (v0.39) with the following parameters: ‘TRAILING:20 and MINLEN:75’ [30]. After adapter trimming and filtering of low-quality reads, 24,308,031 read pairs (89.06%) were retained from the initial reads after YES culture, whereas 24,856,354 read pairs (90.92%) were retained from the initial reads after MEB culture. These read pairs were used for transcript assembly.

### 2.10. Gene Annotation

Genes in the assembled genome were predicted using the FunGAP pipeline (v1.1.1) based on clean RNA-Seq reads [31]. The predicted genes were functionally annotated by a homology-based search against the non-redundant protein database at NCBI using DIAMOND (v2.1.9) with the parameters ‘e-value 0.00001 and –very-sensitive’ [32]. tRNA and rRNA were predicted using tRNAscan-SE (v2.0.12) [33] and Barrnap (v0.9) [34], respectively.

Gene Ontology (GO)-based functional annotation was performed using the DAVID functional annotation tool (https://david.ncifcrf.gov) [35,36], and its *A. alternata* database was used for background comparison. The annotated genes were then assigned to three GO categories (biological processes, cellular components, and molecular functions).

### 2.11. Identification of SM BGCs and Correlation of SM Data from the Predicted SM BGCs in A. alternata KACC 411286 with SMs Produced by the Strain

The antiSMASH (v7.0) program with default settings was used to predict and identify SM BGCs and their signature genes in the assembled genome of *A. alternata* KACC 411286 [37]. To link the SM data from the predicted SM BGCs in *A. alternata* KACC 411286 to SMs produced by the strain, untargeted LC-MS/MS data were subjected to the Global Natural Product Social Molecular Networking (GNPS) [38]. The resulting molecular networks of compounds were visualized using Cytoscape (v3.10.3) [39]. Also, the LC-MS/MS data were analyzed by SIRIUS 4 [40].

### 2.12. Multiple Sequence Alignment and Functional Conservation Analysis of AOH and AOH-Derived Toxin BGCs

For multiple comparisons of the BGCs of AOH and AOH-derived toxins in *A. alternata* KACC 411286 and three other fungal strains, six gene and protein sequences (*pksI*, *omtI*, *moxI*, *aohR*, *sdrI*, and *doxI*) in the cluster for *A. alternata* ATCC 66981, *A. arborescens* EGS 39–128, and *Parastagonospora nodorum* (*P. nodorum*; synonym to *Stagonospora nodorum*) SN15 were retrieved from the genome portal (MycoCosm) of the US Department of Energy (DOE) Joint Genome Institute (JGI) (https://www.mycocosm.jgi.doe.gov/mycocosm/home; accessed on 20 August 2025). The gene sequences from the three closely related fungal strains were then aligned to the genomic data of each strain, which were retrieved from NCBI, using minimap2 [41]. The organization of AOH and AOH-derived toxin biosynthetic genes in the clusters was visualized to compare the gene order, gene size, transcriptional orientation, and intergenic distance.

For functional conservation analysis, the BGCs of AOH and AOH-derived toxins for five other fungal strains (*A. alternata* ATCC 66981, *A. arborescens* EGS 39–128, *A. tenuissima* BMP 0304, *A. mali* BMP 3064, and *P. nodorum* SN15) were retrieved from the genome portal (MycoCosm) of the US DOE JGI. Then, the functional conservation of each gene in the BGCs from *A. alternata* KACC 411286 and the five closely related fungal strains was analyzed by multiple sequence alignments using UniProt. The homology between protein or DNA sequences of each gene in the BGCs of AOH and AOH-derived toxins was determined by the percentages of the sequence alignments. The diagram of the functional conservation analysis was generated using RStudio (v4.3.1) with default parameters. The number of introns within each AOH and AOH-derived toxin biosynthetic gene was analyzed using the FunGAP program (v1.1.1) [31].

### 2.13. Data Availability

The nucleotide sequence data of *A. alternata* KACC 411286 from the Whole Genome Shotgun project have been deposited at DDBJ/ENA/GenBank under the accession number JBUDHX000000000. The version described in this paper is JBUDHX010000000.

The genome and proteome data of *A. alternata* ATCC 66981, *A. arborescens* EGS 39–128, *A. tenuissima* BMP 0304, *A. mali* BMP 0364, and *P. nodorum* SN15 were retrieved from NCBI and the genome portal (MycoCosm) of the US DOE JGI. We selected fungal strains for genome data comparisons based on publicly available information (AOH and AOH-derived toxin producers and genome data availability).

## 3. Results

### 3.1. Effects of Strawberry Extracts on AOH and AME Production by A. alternata KACC 411286

**Figure 1:**
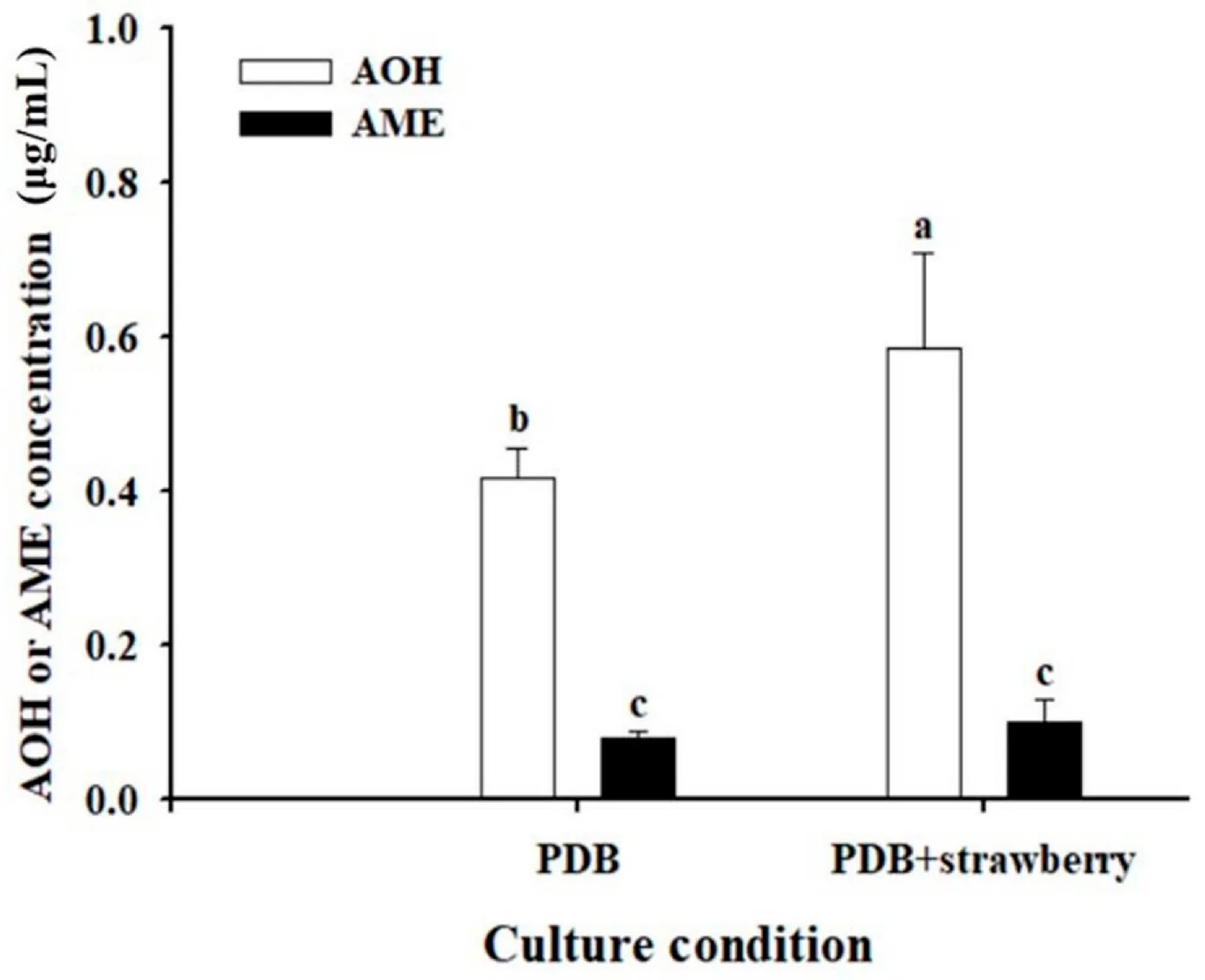
AOH and AME production by *A. alternata* KACC 411286 after culture in PDB or PDB supplemented with strawberry extracts at 25 °C for 5 days under static conditions. The levels of AOH were measured in triplicate. Data are represented as the mean ± standard deviation. Different letters indicate statistically significant differences (*p* < 0.05).

A previous study reported that *A. alternata* ATCC 66981 produced detectable amounts of AOH and AME in mCBD culture containing pieces of tomato compared to a culture without tomato pieces [11]. Thus, we cultured *A. alternata* KACC 411286, previously isolated from strawberry jam [13], in PDB or PDB supplemented with strawberry extract to compare the amounts of AOH and AME produced by the strain. The level of AOH in strawberry extract-enriched PDB (0.59 ± 0.12 μg/mL) was 1.4-fold higher than that in PDB (0.42 ± 0.04 μg/mL) (Figure 1). However, the AME levels were relatively low in both media compared to the AOH levels, and they were not significantly different from each other (0.08 ± 0.01 μg/mL in PDB, 0.10 ± 0.03 μg/mL in PDB plus strawberry extract). This indicates that strawberry extract induced AOH production by *A. alternata* KACC 411286 in PDB.

### 3.2. AOH-Derived Toxins Produced by A. alternata KACC 411286

**Figure 2:**
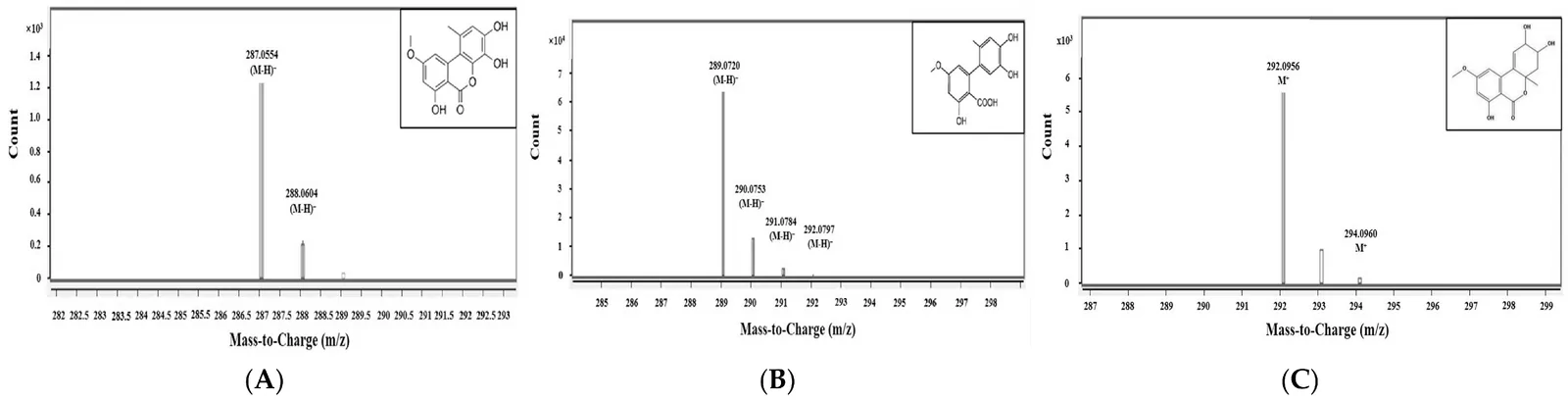
Mass (MS) spectra of 4-OH-AME, ALN, and ALT. (A) MS spectrum for 4-OH-AME, (**B**) MS spectrum for ALN, (**C**) MS spectrum for ALT in culture extracts of *A. alternata* KACC 411286. (Inset) 4-OH-AME structure in (**A**), ALN structure in (**B**), and ALT structure in (**C**).

To investigate whether *A. alternata* KACC 411286 produces AOH-derived toxins such as 4-OH-AME, ALN, and ALT, as well as AOH and AME, an LC/Q-TOF analysis was performed with fungal extracts from PDB supplemented with strawberry extract. As we previously reported, AOH and AME were detected in their MS spectra, and they matched well (scores of 81.76 and 92.68%, respectively) in the METLIN PCDL database, which was connected to the Agilent LC/Q-TOF (Figure S2) [13]. In addition, we detected three other AOH-derived toxins (4-OH-AME, ALN, and ALT) from the fungal extracts (Figure 2 and Figure S2). The mass-to-charge (*m*/*z*) ratio of the most abundant product ion [M-H]^−^ associated with the peak of a compound was 287.0554, which is almost identical to the theoretical molecular mass (288.0629), and its molecular formula was C_15_H_12_O_6_ (81.47% score) (Figure 2A). This suggests that the compound is 4-OH-AME. Also, the *m*/*z* ratio of the major product ion [M-H]^−^ associated with the peak of the second compound was 289.0720, which is almost identical to the theoretical molecular mass (290.0793), and its molecular formula was C_15_H_14_O_6_ (99.63% score) (Figure 2B). This indicates that the second compound is ALN. Finally, the *m*/*z* ratio of the most abundant product ion M^+^ associated with the peak of the third compound was 292.0956, which is almost identical to the theoretical molecular mass (292.0961), and its molecular formula was C_15_H_16_O_6_ (70.42% score) (Figure 2C). This suggests that the third compound is ALT. Thus, we concluded that *A. alternata* KACC 411286 can produce AOH-derived toxins such as 4-OH-AME, ALN, and ALT, as well as AOH and AME.

### 3.3. Relative Gene Expression Analysis by RT-qPCR

**Figure 3:**
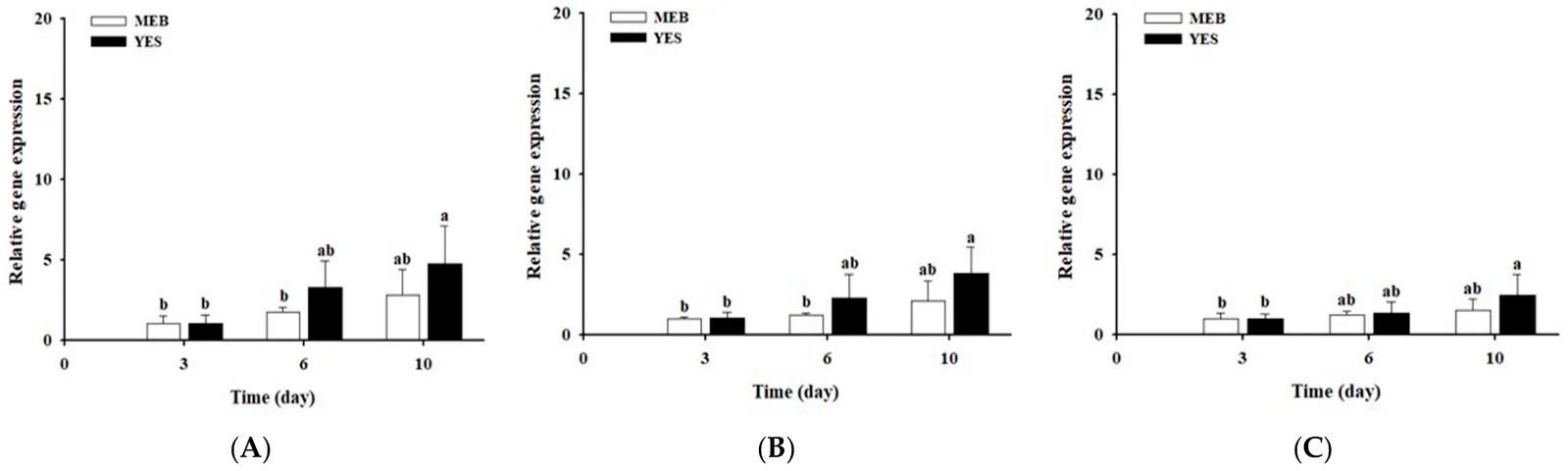
Time course of relative expression levels of three AOH-derived toxin biosynthetic genes in *A. alternaria* KACC 411286 in YES (AOH and AME-supportive) or MEB (AOH and AME non-supportive) medium. Relative expression levels of (**A**) *moxI*, (**B**) *sdrI*, and (**C**) *doxI* in YES or MEB. The expression levels of three genes were measured in triplicate and normalized by that of β-tubulin gene. Data are represented as the mean ± standard deviation. Different letters indicate statistically significant differences (*p* < 0.05).

Since we found that *A. alternata* KACC 411286 can produce three other AOH-derived toxins, the relative expression levels of three ALT biosynthetic genes (*moxI*, *sdrI*, and *doxI*), which are responsible for the production of 4-OH-AME, ALN, and ALT, respectively, in the gene cluster were analyzed by RT-qPCR using *A. alternata* KACC 411286 after culture in YES (AOH and AME-supportive) or MEB (AOH and AME non-supportive) medium. The relative expression levels of all three ALT biosynthetic genes (*moxI*, *sdrI*, and *doxI*) increased gradually with incubation time in both types of media over 10 days (Figure 3). Also, their average expression levels were higher in YES than in MEB after 6 and 10 days although there was no statistically significant difference between the levels in the two media. This is slightly different from our previous results on *pksI* and *omtI*, which are responsible for AOH and AME production, respectively, and whose levels were up-regulated in YES but not in MEB with increasing incubation time [13]. Although the expression levels of *moxI*, *sdrI*, and *doxI* were not statistically different between the two media, they increased progressively with increasing time. Thus, we assume that the transcriptional activation of the genes is closely linked to the metabolic production of 4-OH-AME, ALN, and ALT.

### 3.4. Assembled Genome of A. alternata KACC 411286, and Genome Sequencing Statistics for A. alternata KACC 411286 and 5 Other Fungal Strains

The whole genome of *A. alternata* KACC 411286 was sequenced using the MinION long-read sequencing platform. After genome assembly, in total, 10 contigs were obtained, including the longest contig spanning 7,473,212 bp and the shortest contig spanning 1,851,681 bp (Table S2). The data revealed that *A. alternata* KACC 411286 has a genome size of 34.20 Mb and that the assembled genome has 13,942 putative protein-coding genes (Table S3). It was reported that *Alternaria* spp. usually possess 10 core chromosomes [42] and that *A. alternata* possesses 9 to 11 chromosomes [1,43]. which is in agreement with the 10 chromosomes (equivalent to 10 contigs) of *A. alternata* KACC 411286 in our data (Table S3).

The genome sequencing data of *A. alternata* KACC 411286 and five other fungal strains (four closely related *Alternaria* spp. and one *Parastagonospora* sp.) were compared and analyzed. The genome size of a microorganism usually serves as a crucial indicator of the complexity and functional diversity of its genome [44]. The genome size of the six fungal strains ranged from 33.15 Mb (*A. alternata* ATCC 66981) to 37.21 Mb (*P. nodorum* SN15) (Table 1). It showed that the genome of *A. alternata* KACC 411286 (34.20 Mb) is similar in size to those of *A. arborescens* EGS 39–128 (33.90 Mb), *A. mali* BMP 3064 (34.33 Mb), and *A. tenuissimai* BMP 0304 (34.36 Mb) as well as *A. alternata* ATCC 66981 (33.15 Mb). This is consistent with the fact that they are closely related to each other in the phylogenetic tree, as described previously [1,45].

The GC content of the genome can affect genome stability and structural organization [46]. Table 1 shows that *A. alternata* KACC 411286 has a GC content of 50.97%, which is similar to that of other *A. alternata* strains (50–51%) as reported previously [42,47].

The genome assembly data can be assessed based on the N50 value [48]. The highest N50 value (3.05 Mb) was observed in the genome assembly of *A. alternata* KACC 411286 relative to five other fungal strains, suggesting the highest quality of the genome assembly (Table 1).

The number of protein-coding genes can reflect the metabolic capacity and genetic diversity of fungal strains. The number of predicted protein-coding genes in the *A. alternata* KACC 411286 genome is 13,942 as shown in Table 1. Of the 13,942 protein-coding genes, 13,552 genes (97%) were functionally annotated using the protein database at NCBI and the DIAMOND tool.

**Table 1:** Genome assembly statistics of *A. alternata* KACC 411286 and five other fungal species.

Genome Feature	*A. alternata* KACC 411286	*A. alternata* ATCC 66981	*A. arborescens* EGS 39–128	*A. tenuissima* BMP 0304	*A. mali* BMP 3064	*P. nodorum* SN15
Sequencing technology	Oxford nanopore	Illumina MiSeq	Illumina GA II	Illumina	Illumina	Illumina HiSeq
Genome size (Mb)	34.20	33.15	33.90	33.36	34.33	37.21
Sequencing coverage (x)	76	56	90	NR ^1^	NR	71
Number of contigs	10	215	820	224	2002	31
Number of scaffolds	10	215	820	218	1687	23
Number of scaffolds (≥2 kb)	10	NR	NR	158	1456	NR
N50 (Mb)	3.05	0.39	0.31	0.02	0.30	1.70
GC (%)	50.97	51.5	51.0	51.03	50.84	50.50
Number of protein-coding genes	13,942	NR	NR	NR	NR	17,448

The genome assembly data of five fungal strains were obtained from the previous studies [47,49] and publicly available information at the NCBI and MycoCosm JGI websites. ^1^ NR indicates Not Reported.

### 3.5. Gene Ontology (GO) Classification of Predicted Proteins in A. alternata KACC 411286

GO classification was used to gain functional insights into the annotated genes in the genome of *A. alternata* KACC 411286. All 13,552 functionally annotated proteins in the genome of the strain were analyzed using the DAVID functional annotation tool along with its *A. alternata* database to assign GO terms. Of the 13,552 functionally predicted proteins, 2452 proteins were successfully assigned to three GO categories (401 in biological processes, 1539 in cellular components, and 512 in molecular functions) (Figure S3 and Table S3). The GO classification results showed that genes involved in several functions are predominantly enriched in the genome of *A. alternata* KACC 411286, such as protein synthesis (translation), protein turnover (catabolic) processes, and protein transport in the biological process and cellular component categories, and transcription by RNA polymerase II (RNA binding activity and RNA polymerase II activity) in the molecular function category (Figure S3). This suggests that this strain may be actively involved in essential cellular functions such as RNA synthesis, and protein turnover and synthesis.

### 3.6. SM BGCs in A. alternata KACC 411286 and A. alternata Z7

It has been reported that fungal SMs serve as mediators for interactions between fungi and plant hosts and as pathogenicity factors for fungal survival and defense mechanisms [14]. Generally, fungal SMs are categorized into five main groups: non-ribosomal peptides, polyketides, terpenoids, alkaloids, and ribosomally synthesized and post-translationally modified peptides (RiPPs). A typical SM BGC contains one or more signature genes responsible for core enzymes in the SM biosynthetic pathway, such as non-ribosomal peptide synthetase (NRPS), polyketide synthase (PKS), terpene cyclase (TC), dimethylallyl tryptophan synthase (DMATS), and RiPP precursor synthase, along with tailoring enzymes [50,51]. Moreover, hybrid signature genes are commonly observed, such as NRPS-T1PKS (fusion of NRPS and type 1 PKS) [52]. Hence, to better understand the potential capability for SM production by *A. alternata* KACC 411286, we investigated the predicted SM BGCs in the genome of the fungal strain using the antiSMASH program and compared them with those in *A. alternata* Z7.

A total of 40 SM BGCs were identified in the genome of *A. alternata* KACC 411286. These BGCs include eight NRPS gene clusters, eight PKS gene clusters including one type III PKS, 16 TC gene clusters including two terpene precursor synthases, one fungal RiPP precursor synthase gene cluster, one non-alpha poly-amino acid (NAPAA), and six hybrid gene clusters including NRPS-type I PKS hybrid, an isocyanide synthase-NRPS hybrid, NRPS-indole synthase hybrid, and NRPS-metallophore hybrid (Table 2 and Table S5). These data also showed that the number of SMs in *A. alternata* KACC 411286 is higher than the 31 SMs in *A. alternata* Z7 (a tangerine pathotype) although they have similar genome sizes (approximately 34.2 Mb) (Table 1 and Table S5) [42]. Interestingly, *A. alternata* KACC 411286 possesses a higher number of TC gene clusters (16) than *A. alternata* Z7 (7) (Table S5).

Also, our SM BGC data revealed that the genome of *A. alternata* KACC 411286 has a BGC of terpestacin, which is known to have anti-HIV and anticancer potential [53,54]. In addition, we found that *A. alternata* KACC 411286 contains an equisetin BGC (Table 2), whose product is a tetramic acid derivative produced mainly by *Fusarium equisti*. One previous study reported that equisetin has inhibitory effects on HIV-1 integrase activity and a broad spectrum of anti-fungal and anti-bacterial activity against plant pathogens, as well as herbicidal activity against weeds [55]. Also, a BGC of another tetramic acid derivative TeA was not detected in *A. alternata* KACC 411286, which is consistent with the results of no TeA production by the strain from our previous study [13]. In addition, the genome of *A. alternata* KACC 411286 contains metachelin C, A, A-CE, and B BGCs (Table 2), which can synthesize the iron-chelating siderophore to overcome host plant immunity [56]. Moreover, we detected the squalestatin S1 BGC in the *A. alternata* KACC 411286 genome, which can be used as a cholesterol-lowering agent for the control of cholesterol biosynthesis in mammals (Table 2) [57].

In addition, we identified four other mycotoxin BGCs (PR-toxin, alternapyrone, betaenone, chaetoglobosin P) as well as the AOH BGC in the genome of *A. alternata* KACC 411286 (Table 2). It is known that PR-toxin is produced mainly by *Penicillium* spp. such as *P. roqueforti* in Roquefort cheese and that it causes cytotoxicity, immunotoxicity, and carcinogenicity in humans and animals [58]. Another mycotoxin, alternapyrone, is frequently produced by *Alternaria solani* on tomato and potato, resulting in early blight disease [59]. Notably, alternapyrone was isolated from *P. nodorum* SN15, which is known to produce AOH [60,61]. This may suggest that *P. nodorum* SN15 shares certain mycotoxin BGCs with *A. alternata* although they are relatively distantly related to each other. Also, a previous study reported that BGCs for the production of some other mycotoxins such as cercosporin, dothistromin, and versicolorin B were identified in the genome of *A. alternata* Y784-BC03 [62]. However, we did not detect these SM BGCs in the genome of *A. alternata* KACC 411286.

**Figure 4:**
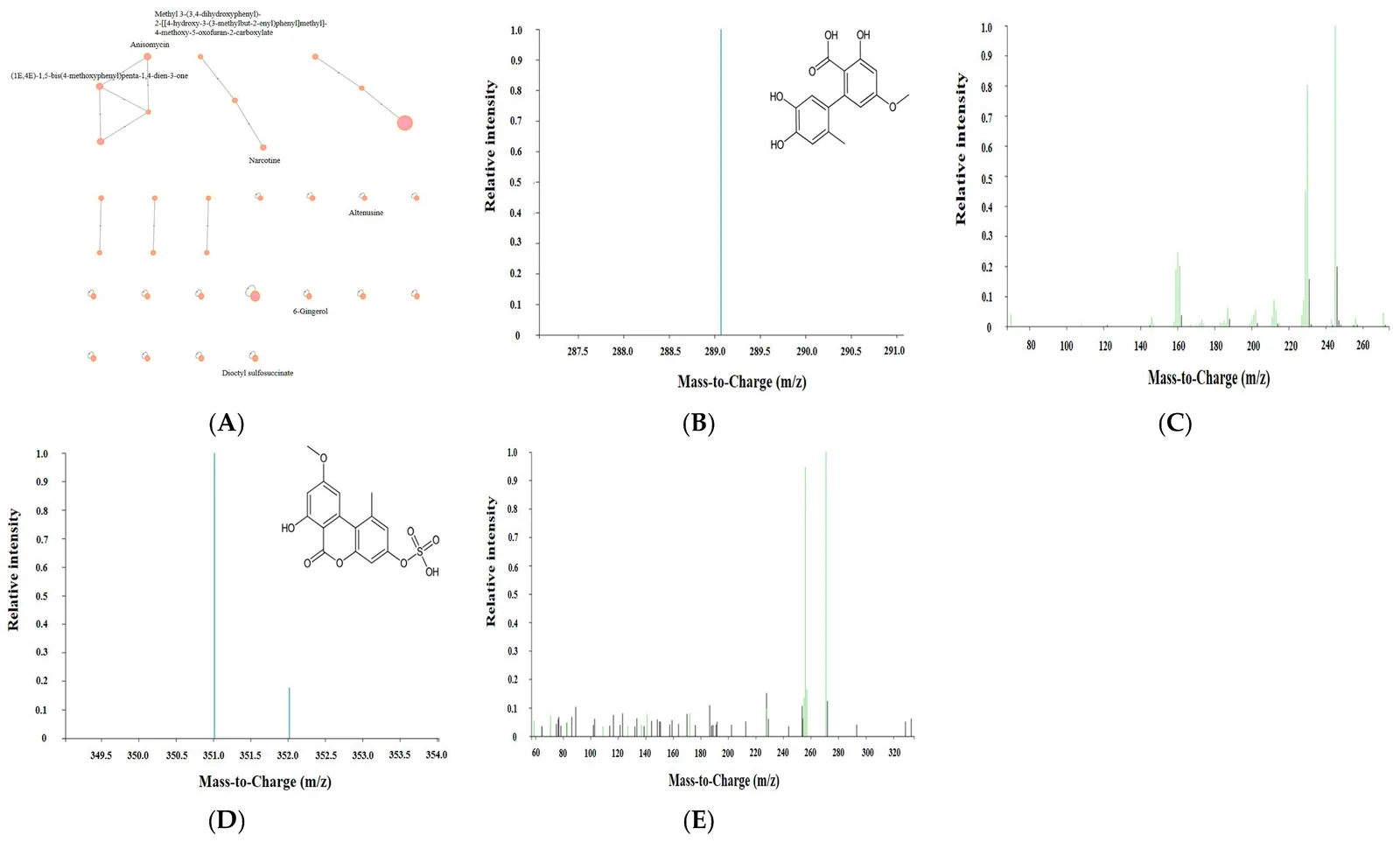
Untargeted network, and MS and MS/MS spectra of SMs identified in culture extracts of *A. alternaria* KACC 411286 using GNPS and SIRUS4. (**A**) Molecular network of seven SMs identified in the culture extracts using the GNPS repository, and (**B**) MS and (**C**) MS/MS spectra of ALN, and (**D**) MS and (**E**) MS/MS spectra of alternariol 5-O-methyl ether-4′-O-sulfate identified in the culture extracts using SIRIUS4. Each node represents the precursor ion of each SM, and its size indicates the relative intensity of the precursor ion in (**A**). (Inset) ALN structure in (**B**) and alternariol 5-O-methyl ether-4′-O-sulfate structure in (**D**).

On the other hand, we used untargeted LC-MS/MS data on SMs produced by *A. alternata* KACC 411286 to link the SM data produced by the strain to SMs from its predicted SM BGCs. Seven SMs including ALN were detected from a database in the GNPS repository (Figure 4A, Table S6), in which some unknown SMs can be identified based on LC-MS/MS data of known SMs. The networks of the seven SMs are visualized in Figure 4A. Also, we analyzed the LC-MS/MS data on SMs produced by *A. alternata* KACC 411286 using SIRIUS 4, which is known to be more effective for the identification of small SMs (<600 Da). Only four SMs including ALN (100% score) and betaenone C (100% score) were identified from a total of 62 SMs, which were detected using the LC-MS/MS database at SIRIUS4 (Figure 4B,C and Figure S4, Table S6); the *m*/*z* ratio of the most abundant product ion [M-H]^−^ (289.072) was almost identical to the molecular mass of ALN (M = 290.0793), and the *m*/*z* ratio of [M-H]^−^ (365.234) was almost identical to the molecular mass of betaenone C (M = 366.2406) with MS/MS profiles similar to that of each compound. Notably, alternariol 5-O-methyl ether-4′-O-sulfate, presumably a metabolite of AOH, AME, or 4-OH-AME, was detected (93.9% score, [M-H]^−^ = 351.018, M = 352.0253) (Figure 4D,E). It is consistent with a report that AOH or AME can form its sulfate conjugate for detoxification in *A. alternata* [4,63,64]. These data indicate that two (AOH-derivatives [AOH, AME, 4-OH-AME, and ALN], betaenone C) out of the 13 predicted major SMs that can be produced from the 40 SM BGCs, which were detected in the genome of *A. alternata* KACC 411286 as shown in Table 2, were actively produced by the strain in the PDB medium.

### 3.7. Multiple Sequence Comparison and Functional Conservation Analysis of AOH-Derived Toxin BGCs in A. alternata KACC 411286 and 5 Other Fungal Strains

**Figure 5:**
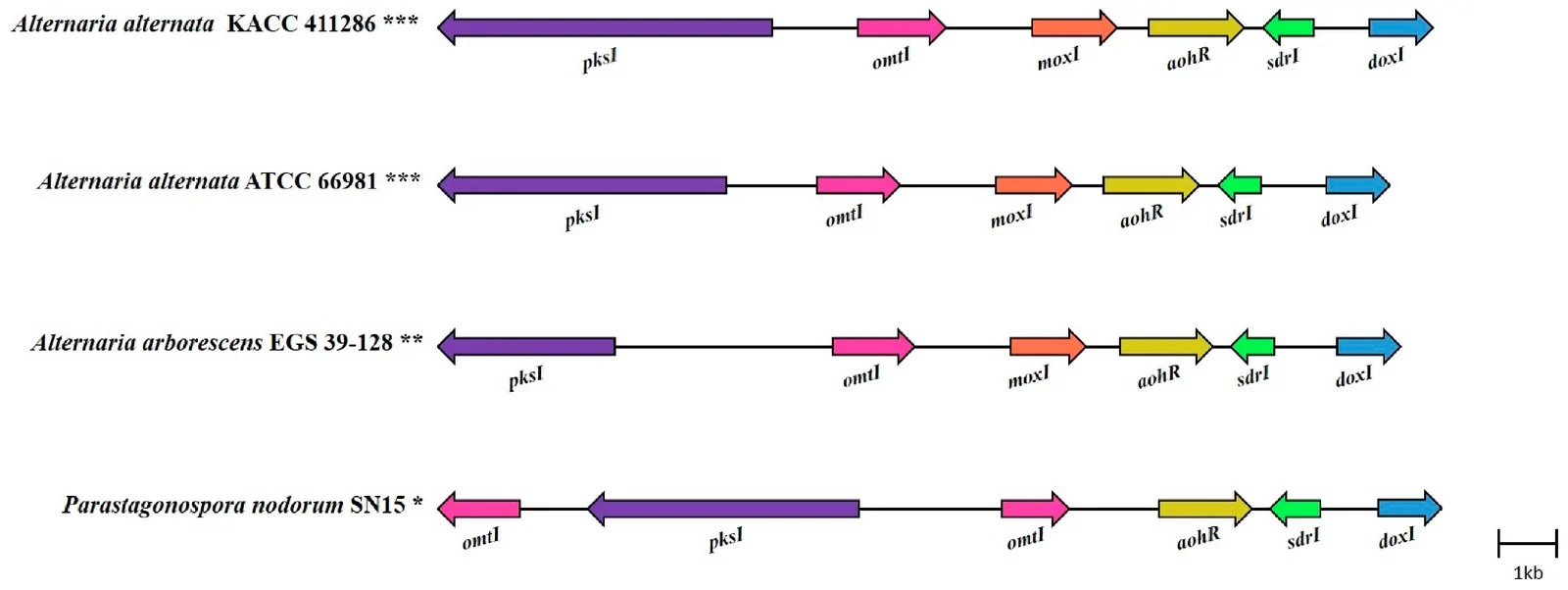
Comparative analysis of ALT BGCs in *A. alternaria* KACC 411286 and three other fungal strains. Gene names are shown below the gene cluster. Arrows indicate the orientation of transcription of the genes. Homologous genes are represented in the same color among four different fungal strains. The asterisks (*, **, and ***) indicate AOH-producing strains, AOH- and AME-producing strains, and five AOH-derived toxin-producing strains, respectively, based on data from the current study and previous studies [11,61,65,66,67].

Previously, a study described the gene clusters responsible for the biosynthesis of five AOH-derived toxins (AOH, AME, 4-OH-AME, ALN, and ALT) in *P. nodorum* and several *Alternaria* spp. such as *A. alternata*, *A. arborescens*, and *A. tenuissima* [11]. This showed that the ALT BGCs in the *Alternaria* spp. comprise all six genes, of which one gene encodes a transcription factor (*aohR*) and five genes encode biosynthetic enzymes (*pksI*, *omtI*, *moxI*, *sdrI*, and *doxI*), whereas the BGC in *P. nodorum* possesses five genes, excluding *moxI*. In this study, we identified the functional and complete ALT BGC (17.5 kb) in the genome of *A. alternata* KACC 411286. Hence, we compared the ALT BGC in *A. alternata* KACC 411286 with those from three other fungal strains, including *P. nodorum* SN15. The analysis revealed that three *Alternaria* spp. including *A. alternata* KACC 411286 possess all six biosynthetic genes in the gene cluster, which are responsible for ALT biosynthesis, while *P. nodorum* SN15 possesses only five ALT biosynthetic genes, excluding *moxI* in the incomplete gene cluster (Figure 5). These data may suggest why the two *Alternaria alternata* are ALT producers, while *P. nodorum* SN15 is only an AOH producer; although it was reported that *A. arborescens* EGS 39–128 (=BMP 0308) produces AOH and AME, we assume that it could produce other AOH-derived toxins [65,66].

Also, the order of ALT biosynthetic genes in the gene cluster in *A. alternata* KACC 411286 was the same as in the two *Alternaria* spp. (the all-four AOH-derived toxins-producing *A. alternata* ATCC 66981, AOH- and AME-producing *A. arborescens* EGS 39–128), and the transcriptional orientation of the ALT biosynthetic genes was also conserved among the three *Alternaria* spp. (Figure 5). These data suggest that the organization of the ALT BGCs is highly conserved in the AOH and AME producers. However, in *P. nodorum* SN15, five ALT biosynthetic genes, excluding *moxI*, were detected along with another copy of *omtI* as described above (Figure 5). Interestingly, except for the absence of *moxI* and the presence of the *omtI* gene downstream of *pksI*, the organization of the ALT BGC in *P. nodorum* SN15 is conserved with three AOH- and AME-producing *Alternaria* strains. Given that *P. nodorum* SN15 is phylogenetically distant from the three other *Alternaria* spp., the ALT BGC in *P. nodorum* SN15 may have undergone genomic divergence by genetic loss due to chromosomal rearrangements or the insertion of transposable elements during its evolution.

In addition, we analyzed the size of each ALT biosynthetic gene and its corresponding protein and the number of introns within the gene in *A. alternata* KACC 411286 using the FunGAP program and compared them with those from *A. alternata* ATCC 66981. The size of the same ALT biosynthetic gene and its corresponding protein in *A. alternata* KACC 411286 was similar to that in *A. alternata* ATCC 66981. The number of introns in the same gene was identical between the two strains except for *omtI* (Table S7).

**Figure 6:**
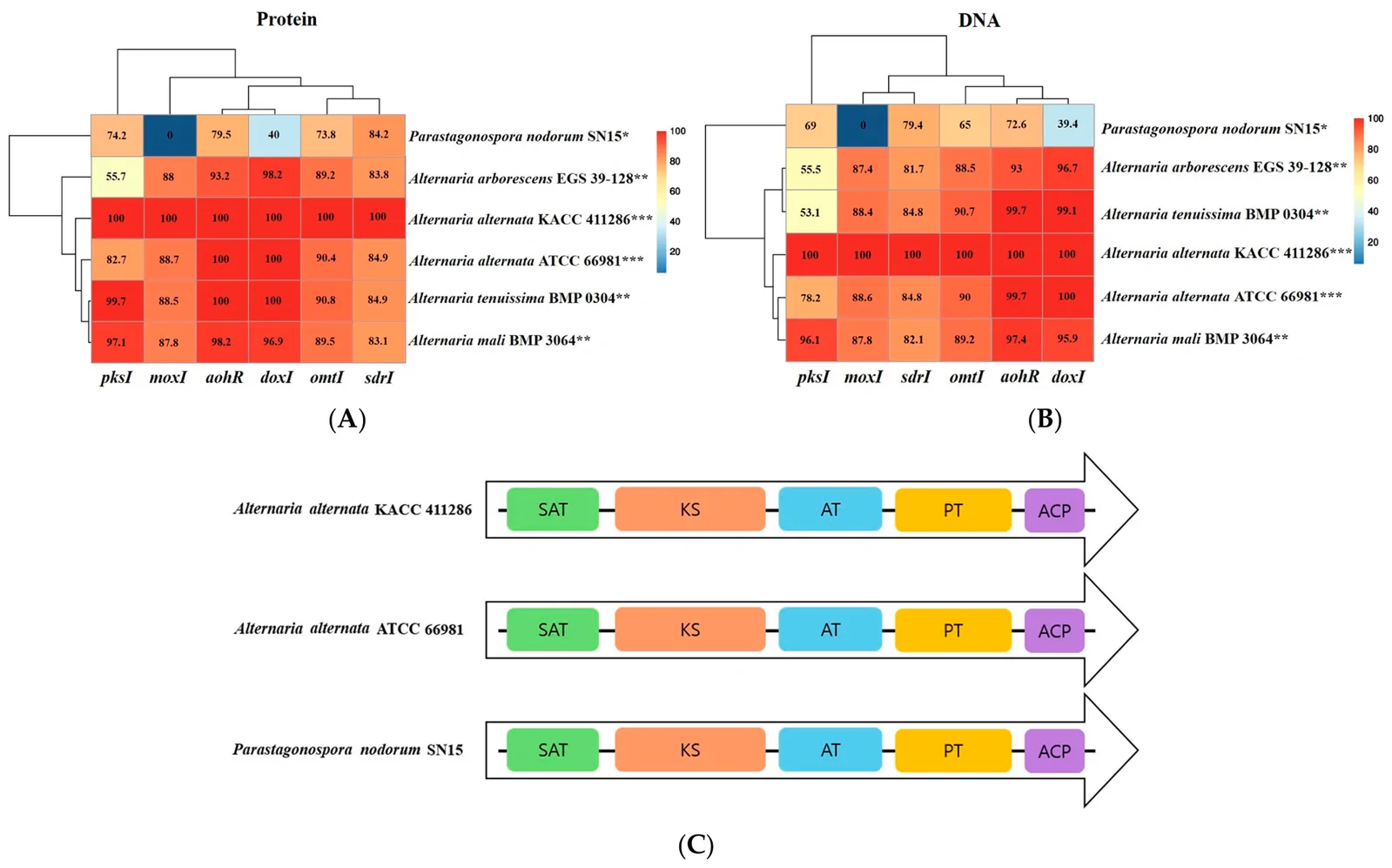
Functional conservation analysis of ALT BGCs and PksI domains in *A. alternaria* KACC 411286 and other fungal strains. The percent identity among (**A**) the amino acid and (**B**) DNA sequences from the homologous genes is shown as indicated by the color key when it is based on 100% sequence identity of each gene in *A. alternaria* KACC 411286, and (**C**) 5 PksI domains in PksI between *A. alternaria* KACC 411286 and 2 fungal strains are shown. The asterisks (*, **, and ***) represent an AOH-producing strain, AOH- and AME-producing strains, and five AOH-derived toxin-producing strains, respectively, in (**A**,**B**). The direction of the arrow indicates protein translation from N-terminal sequence in (**C**). Abbreviations: SAT, starter unit:ACP transacylase; KS, ketosynthase; AT, acyltransferase; PT, product template; ACP, acyl carrier protein.

Comprehensive functional conservation analyses based on protein and DNA sequences were carried out on 6 ALT biosynthetic genes in the gene clusters from *A. alternata* KACC 411286 and five other fungal strains. The analysis revealed that each ALT biosynthetic gene in *A. alternata* KACC 411286 shares a high percentage of sequence identity (above 78% identity) at both amino acid and DNA levels with the corresponding gene in four other *Alternaria* spp. except *pksI* in *A. arborescens* EGS 39–128 (55% identity at both the protein and DNA levels) and *pksI* in *A. tenuissima* BMP 0304 (53% at DNA level), which exhibit a slightly lower percentage of sequence identity (Figure 6A,B). However, four genes including *pksI* (*pksI*, *omtI*, *sdrI*, and *aohR*) in *P. nodorum* SN15 share a high percentage of sequence identity at both the amino acid and DNA levels (above 73.8% identity at the protein level, above 65% identity at the DNA level) with the corresponding gene in *A. alternata* KACC 411286, whereas two other genes (*moxI* and *doxI*) in *P. nodorum* SN15 share a low percentage of identity (below 40% identity) at both the protein and DNA levels with the corresponding genes in *A. alternata* KACC 411286 (Figure 6A,B). These results may explain why *P. nodorum* SN15 only produces AOH, but no AME production in the strain remains yet to be determined [61]. Overall, our data demonstrate that *A. alternata* KACC 411286 shares highly homologous ALT biosynthetic enzymes for the production of five AOH-derived toxins (AOH, AME, 4-OH-AME, ALN, and ALT) with the four closely related *Alternaria* spp., of which *A. alternata* ATCC 66981 is known to be an all-five-toxin producer and three other strains are known to be AOH and AME producers [11,65,66,67]. It would be necessary to verify production of the three other AOH-derived toxins by the strains in future studies.

In addition, we analyzed the organization of the domains of PksI in *A. alternata* KACC 411286 and compared it with those in *A. alternata* ATCC 66981 and *P. nodorum* SN15. The data showed that the three fungal strains share the conserved organization of the PksI domains, which is typical of fungal iterative type I PKS and consists of 5 domains (starter unit:ACP transacylase domain [SAT], ketosynthase domain [KS], acyltransferase domain [AT], product template domain [PT], acyl carrier protein domain [ACP]). These data may suggest that the three strains have evolved a convergent mechanism for AOH production.

## 4. Discussion

It has been known that filamentous fungi are prolific producers of bioactive SMs. SMs play an essential role in fungal survival in their ecological niche [14]. Although some of these SMs are used as drug leads beneficial to humans, such as the antibiotic penicillin and the cholesterol-lowering agent lovastatin, many of them are harmful mycotoxins, including aflatoxins, patulin, and AOH [68].

We previously documented AOH- and AME-producing *A. alternata* KACC 411286 isolated from strawberry jam [13]. Much is unknown about SM BGCs, including the AOH-derived toxin BGC in *A. alternata*. Whole-genome sequencing and comparative analysis of *A. alternata* can help understand the molecular mechanisms underlying SM biosynthesis in closely related *Alternaria* species. Thus, in the present study, to gain valuable knowledge about the biosynthesis of other SMs, as well as AOH-derived toxins in *A. alternata* KACC 411286, its whole genome was sequenced, and BGCs of SMs, including AOH-derived toxins, were analyzed.

The genome sequencing data of *A. alternata* KACC 411286 showed that the strain has 10 chromosomes, all of which are larger than approximately 2 Mb in size. It has been reported that the genes responsible for the production of host-specific toxins are located on conditionally dispensable chromosomes (CDCs), which are mostly small chromosomes less than 2.0 Mb and are not required for fungal growth but for its pathogenicity against plant hosts [3,69,70]. It seems that *A. alternata* KACC 411286 is not able to produce host-specific toxins such as AAL- ACT-, and AF-toxins because it is not likely to possess CDCs carrying their biosynthetic genes. This is strengthened by the fact that *A. alternata* Z7, which is a tangerine pathotype, has two additional CDCs on which ACT-toxin biosynthetic genes reside, in addition to 10 chromosomes [42]. Moreover, the previous study described that an endophytic *A. alternata* JS-0527 has 10 essential chromosomes without CDCs, which is in accordance with our results. Our genome results also showed that although *A. alternata* KACC 411286 has a similar genome size (approximately 34.2 Mb) to *A. alternata* Z7 (a tangerine pathotype), the number of SMs in *A. alternata* KACC 411286 (40) is higher than that in *A. alternata* Z7 (31). This may suggest that *A. alternata* KACC 411286 can survive in various ecological habitats by producing a higher number of SMs than *A. alternata* Z7 even though the latter can produce tangerine-specific ACT-toxin.

In this study, we observed 1.4-fold higher amount of AOH production by *A. alternata* KACC 411286, which was isolated from strawberry jam, in strawberry extract-enriched PDB compared to that in PDB. One previous study documented that both AOH and AME were detected at significant levels from extracts of mCBD cultures containing tomato fragments of *A. alternata* ATCC 66981, while neither compound was detectable in mCBD culture extracts of the strain, even though the fungal strain was isolated from peanuts [11]. These data may suggest that both AOH and AME are virulence factors against plants and that the production of non-host-specific toxins such as AOH and AME is stimulated by various plants since it has been reported that *A. alternata* has a broad host range and infects a variety of agricultural crops, including fruits and vegetables [3,11].

An LC/Q-TOF analysis verified the production of three additional AOH-derived toxins (4-OH-AME, ALN, and ALT) as well as AOH and AME by *A. alternata* KACC 411286 under PDB conditions. This indicates that the fungal strain possesses the metabolic capacity to synthesize multiple polyketide-derived toxins in response to environmental conditions. In particular, it was reported that ALN has antifungal activity [71], and ALN production by *A. alternata* KACC 411286 may assist its survival against highly competitive environments, including against other fungi. Also, RT-qPCR analysis showed different regulation of the genes responsible for the production of AOH-derived toxins in the strain under AOH-supportive and non-supportive conditions. Previously, we reported that the relative expression levels of *pksI* and *omtI* (genes responsible for AOH and AME syntheses, respectively) in *A. alternata* KACC 411286 were up-regulated in YES medium (AOH- and AME-conducive), while they were not in MEB medium (AOH and AME non-conducive) [13]. In this study, the expression levels of three other genes (*moxI*, *sdrI*, and *doxI*), which are responsible for the syntheses of OH-AME, ALN, and ALT, respectively, were slightly different from those of *pksI* and *omtI*. The reason for this discrepancy remains to be resolved.

In addition, LC-MS/MS and GNPS/SIRIUS4-based analyses of SMs produced by *A. alternata* KACC 411286 revealed that many of the SMs predicted by antiSMASH were not identified among the compounds in the GNPS repository or those predicted by SIRIUS. One of the possible reasons is that the expression of the SM BGCs may be silent in the PDB culture. The antiSMASH analysis suggests that this strain is a potential producer of a wide array of bioactive SMs. Thus, it would be of great interest to activate these cryptic SM BGCs in this fungal strain in future studies. Interestingly, the fungal strain produced alternariol 5-O-methyl ether-4′-O-sulfate, presumably a metabolite of AOH, AME, or 4-OH-AME, under PDB culture conditions. Previously, Lou and collaborators reported that the compound was produced by *Alternaria* sp. as described above [63]. Also, Soukup and co-workers documented that an *A. alternata* strain produced several sulfate conjugates of AOH and AME (AOH 3-sulfate and AOH 9-sulfate from AOH, AME 3-sulfate from AME) on potato dextrose agar (PDA) media [64]. Moreover, two earlier studies described that zearalenone (ZEN) and deoxynivalenol (DON) were converted to ZEN 14-sulfate and DON 3-sulfate, respectively, by the fungal strain *Sphaerodes mycoparasitica*, which are less toxic than the parental compounds, for detoxification [72,73]. Thus, we assume that *A. alternata* KACC 411286 might have converted AOH or AME into their sulfate conjugates, presumably less toxic or non-toxic metabolites, for self-protection via a biotransformation process.

Also, our data showed that the ALT BGC in *A. alternata* KACC 411286 (17.5 kb) is highly conserved with that in *A. alternata* ATCC66981 (15 kb) [11]. However, two copies of *omtI* (located downstream and upstream of *pksI*) were detected in the wheat pathogen *P. nodorum* SN15 as previously described [61,68]. Considering the organization of the ALT BGC and the higher protein sequence identity of the upstream OmtI (73.8%) to OmtI in *A. alternata* KACC 411286 than the downstream OmtI (33.8%), *omtI*, which is located upstream of *pksI* in *P. nodorum* SN15, is likely to be an ortholog of *omtI* in AOH- and AME- producing *Alternaria* strains. In a previous study, AOH production by *P. nodorum* SN15 correlated well with the expression of the SnPKS19 gene in the strain, and the expression of the SnPKS19 gene was co-regulated with *aohR*, *sdrI*, and *omtI*, which is located in the vicinity of *aohR* [61]. Thus, these data support the hypothesis that *omtI* upstream of *pksI* in *P. nodorum* SN15 is responsible for AOH production.

Fungal iterative type I PKS can be divided into non-reducing (NR-), partially reducing (PR-), and highly reducing (HR-) PKS [74]. The PksI from *A. alternata* KACC 411286 belongs to NR-PKS, which consists of multiple catalytic domains such as SAT, KS, AT, PT, and ACP. It is consistent with a previous study which described that NR-PKS is typically involved in the biosynthesis of aromatic polyketides instead of aliphatic compounds [75]. When we compared PksI domains in *A. alternata* KACC 411286 with those in *A. alternata* ATCC 66981 and *P. nodorum* SN15, the results showed that the organization of the PksI domains is identical and highly conserved between them. In particular, the PksI domains in the three strains, including *A. alternata* KACC 411286 lack the C-terminal thioesterase (TE) releasing domain that is required for final product release by either Claisen cyclization or hydrolysis [75,76]. It is likely that the PksIs in the three fungal strains have evolved the ability to release AOH via Claisen cyclization or hydrolysis without the C-terminal TE releasing domain.

Additionally, our functional conservation analysis data showed that high sequence similarity was observed between two *A. alternata* strains, *A. tenuissima* BMP 0304, and *A. mali* 3064. One previous study reported that the *A. alternata* genome exhibited more than 98% average nucleotide identity (ANI) to *A. tenuissima* and *A. mali* genomes [42]. This might support our data. However, unexpectedly, PksI in *P. nodorum* SN15 shares higher sequence identity at both amino acid and DNA levels (69 and 74.2%, respectively) with that in *A. alternata* KACC 411286 than with that in *A. arborescens* EGS 39–128 (55.5 and 55.7%, respectively). This may suggest that PksI in *P. nodorum* shares a common ancestry with *A. alternata* strains.

Our results showed that *A. alternata* KACC 411286 possesses the same number of TC gene clusters (16) as NRPS and PKS gene clusters among the five main SM groups. This is slightly different from a previous study, which showed that NRPS and PKS gene clusters were the most prevalent groups in various *Alternaria* stains, including *A. alternata* Z7 shown in Table S5 [77]. Also, it has been reported that terpenes are widely used in the cosmetics, pharmaceutical, and food industries due to their distinct aromatic, antimicrobial, and antioxidant properties [78,79]. It would be interesting to identify the structures of the terpenes that can be produced by *A. alternata* KACC 411286 in the future. Also, we found one fungal RiPP precursor synthase BGC in the genome of *A. alternata* KACC 411286. Fungal RiPPs are known to have various biological activities such as phytotoxic, nematocidal, and antimitotic effects [80]. In particular, a previous study recently reported that a derivative of asperigimycin (a class of RiPPs) from *Aspergillus flavus* exhibited anticancer activity [81]. Thus, it would be valuable to purify the RiPPs that can be produced by *A. alternata* KACC 411286 and elucidate their chemical structures in future studies.

We also found that the genome of *A. alternata* KACC 411286 possesses a BGC of non-proteinogenic amino acid, called non-alpha poly-amino acid (NAPAA), which is known to have antimicrobial properties that can be used for biotechnological applications [82]. For future studies, the isolation, purification, and structure elucidation of the NAPAA that can be produced by *A. alternata* KACC 411286 could also be a valuable next step.

In addition, in this study, we identified some predicted BGCs for the production of potentially beneficial bioactive SMs such as terpestacin, equisetin, and squalestatin S1 in *A. alternata* KACC 411286 using the antiSMASH online tool. However, no transcriptomic data or LC-MS/MS data on the activation of these beneficial SM BGCs are provided in this study. We assume that these BGCs are silent under the PDB culture conditions. Their functional validation is reserved for future investigation. Also, the program showed that two-thirds (27) of the SM BGCs detected in the genome of the strain (40) produce unidentified SMs. In addition, we attempted to link the SM data from SM BGCs in *A. alternata* KACC 411286 via antiSMASH to SMs produced by the strain using untargeted LC-MS/MS data via GNPS/SIRIUS4. We identified only a small number of SMs, including ALN. It would be necessary to characterize the BGCs responsible for unknown SMs potentially produced by *A. alternata* KACC 411286 in future studies. Our study could pave the way for the discovery of novel bioactive natural products with potentially valuable applications.

On the other hand, in the current study, we verified the production of five AOH-derived toxins by six enzymes encoded by their corresponding genes within the ALT BGC in the genome of *A. alternata* KACC 411286, which was isolated from strawberry jam. Since *A. alternata* can infect a wide range of plant hosts, various fruits, including strawberries, apples, and tomatoes, could commonly be contaminated with multiple mycotoxins such as AOH and AME. Our study provides a genetic basis for the development of effective strategies to mitigate the contamination of fruits with multiple mycotoxins.

## 5. Conclusions

In the present study, we (1) identified three additional AOH-derived toxins (4-OH-AME, ALN, and ALT) as well as AOH and AME produced by *A. alternata* KACC 411286, (2) found increases in the relative expression of the three genes in the PDB culture of the strain with incubation time, (3) identified 40 SM BGCs and 6 ALT biosynthetic genes within the BGC in the strain, and (4) found conserved ALT BGCs between *A. alternata* KACC 411286 and other closely related fungal strains by comparative genomic analysis. These results will improve our understanding of the biosynthesis of SMs, including five AOH-derived toxins in *A. alternata* KACC 411286. Moreover, we identified several BGCs for the production of potentially beneficial bioactive SMs such as terpestacin (an anticancer compound), equisetin (an antimicrobial compound), and squalestatin S1 (a cholesterol-lowering agent) in *A. alternata* KACC 411286. Our findings could facilitate further verifying the production of these potentially beneficial products, finding uncharacterized beneficial novel natural products, and exploring their biosynthetic mechanisms beyond that of ALT in *Alternaria* species.

## Figures and Tables

**Table 2:** Classification and prediction of 40 SM BGCs and 13 major SMs identified in the genome of *A. alternata* KACC 411286.

Type	Number of SM Gene Cluster	Contig Number	Length (bp)	Most Similar Known Cluster
T1PKS ^1^	3	1	67,825	alternapyrone
			66,583	scytalone/1,3,6,8-tetrahydroxynaphthalene
			65,795	alternariol
	1	2	68,260	-
	2	3	67,919	abscisic acid
			66,886	-
	1	9	51,206	betaenone C/probetaenone I/stemphyloxin II/compound 4/compound 6/compound 7/compound 9/compound 3/dehydroprobetaenone I/compound 5
NRPS-T1PKS	1	2	72,462	equisetin
	1	4	72,257	-
	1	6	91,127	-
T3PKS	1	5	61,159	-
NRPS	2	1	65,929	metachelin C/metachelin A/metachelin A-CE/metachelin B/dimerumic acid 11-mannoside/dimerumic acid
			75,485	-
	1	9	82,039	-
NRPS-like	2	7	63,256	-
			63,755	-
	1	8	63,274	-
	1	9	63,352	-
	1	10	63,848	choline
NRP-metallo- phore-NRPS	1	1	85,077	-
NRPS-indole	1	8	80,384	-
Isocyanide-NRP	1	4	71,471	-
NAPAA ^2^	1	7	49,292	-
Terpene	2	1	32,314	-
			30,854	-
	6	2	31,438	-
			31,567	-
			30,779	-
			32,360	clavaric acid
			31,482	-
			32,698	-
	4	4	32,206	terpestacin/preterpestacin 3/preterpestacin 2/preterpestacin 1
			31,617	squalestatin S1
			31,086	chaetoglobosin P
			31,897	-
	1	8	32,202	-
	1	9	31,105	PR-toxin
Terpene precursor	1	2	31,101	-
	1	3	31,438	-
Fungal-RiPP	1	9	52,752	-
Total	40	-	-	-

^1^ T1PKS indicates type 1 PKS. ^2^ NAPAA represents non-alpha poly-amino acid.

## Data Availability

All data generated or analyzed during this study are included in this published article and Supplementary Materials.

## Supplementary Materials

The following supporting information can be downloaded at: https://www.mdpi.com/article/10.3390/jof12090634/s1, Figure S1: Standard curves of AOH and AME standard solutions for quantification of (A) AOH and (B) AME by HPLC-UVD; Figure S2: Extracted ion chromatograms (EIC) and mass (MS) spectra of AOH, AME, 4-OH-AME, ALN, and ALT; Figure S3: Gene Ontology (GO) classification of predicted proteins in *A. alternaria* KACC 411286; Figure S4: MS and MS/MS spectra of betaenone C identified in the culture extracts of *A. alternaria* KACC 411286 using SIRUS4; Table S1: Primer sequences for RT-qPCR analysis of AOH-derived toxin biosynthetic genes; Table S2: Lengths of ten contigs from the draft genome assembly for *A. alternata* KACC 411286; Table S3: Genome assembly data for *A. alternata* KACC 411286; Table S4: Detailed information of Gene Ontology (GO) classification of predicted proteins in *A. alternata* KACC 411286; Table S5: Different types of SM BGCs detected in genomes of *A. alternata* KACC 411286 and *A. alternata* Z7; Table S6: Identification of SMs produced by *A. alternata* KACC 411296 using LC-MS/MS database at GNPS site or SIRIUS4; Table S7: Functions of six ALT biosynthetic genes and comparison of the size of each gene and the number of introns in the gene from *A. alternata* KACC 411286 with those from *A. alternata* ATCC 66981.

## Abbreviations

The following abbreviations are used in this manuscript:

ALN	Altenusin
ALT	Altenuene
AME	Alternariol monomethyl ether
AOH	Alternariol
BGC	Biosynthetic gene cluster
CDC	Conditionally dispensable chromosome
SM	Secondary metabolite
TeA	Tenuazonic acid
