# Supplementary material for: Comparative Whole-Genome Sequence Analysis of Alternaria alternata KACC 411286 That Produces Alternariol-Derived Toxins and Its Secondary Metabolite Biosynthetic Potential
Source: J Fungi (Basel). 2026 Aug 24;12(9):634. doi: 10.3390/jof12090634 (PMC13608438; doi:10.3390/jof12090634)
Supplement: Supplementary file 1 [file jof-12-00634-s001.zip › jof-4467341-supplementary.pdf]

Supplementary Figures

A.

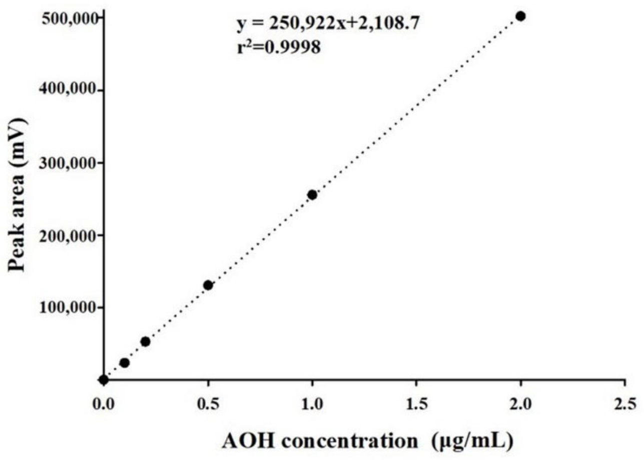

B.

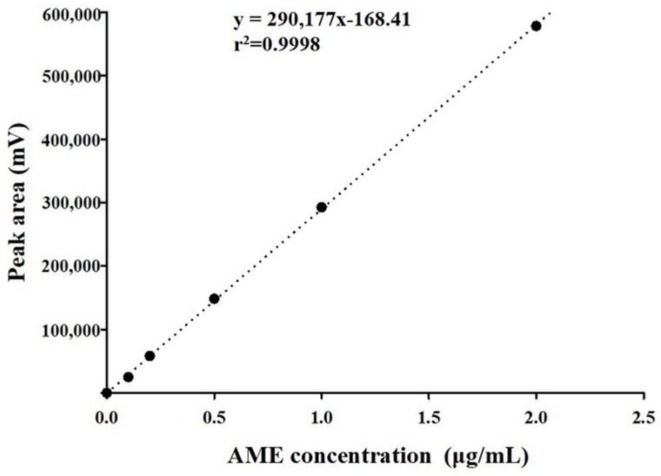

Figure S1. Standard curves of AOH and AME standard solutions for quantification of (A) AOH and (B) AME by HPLC-UVD. A series of AOH or AME standard solutions were prepared in the range of 0.1, 0.2, 0.5, 1.0, and 2.0 μg/mL. Each solution was injected into HPLC-UVD in triplicate.

A.

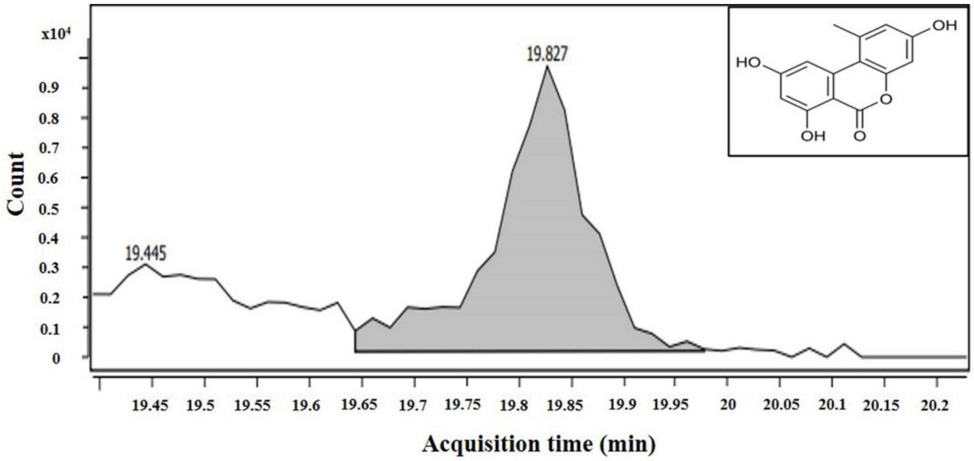
x10^4
0.9
0.8
0.7
0.6
0.5
0.4
0.3
0.2
0.1
0
Count
19.827
19.445
HO
OH
OH
O
O
19.45 19.5 19.55 19.6 19.65 19.7 19.75 19.8 19.85 19.9 19.95 20 20.05 20.1 20.15 20.2
Acquisition time (min)

B.

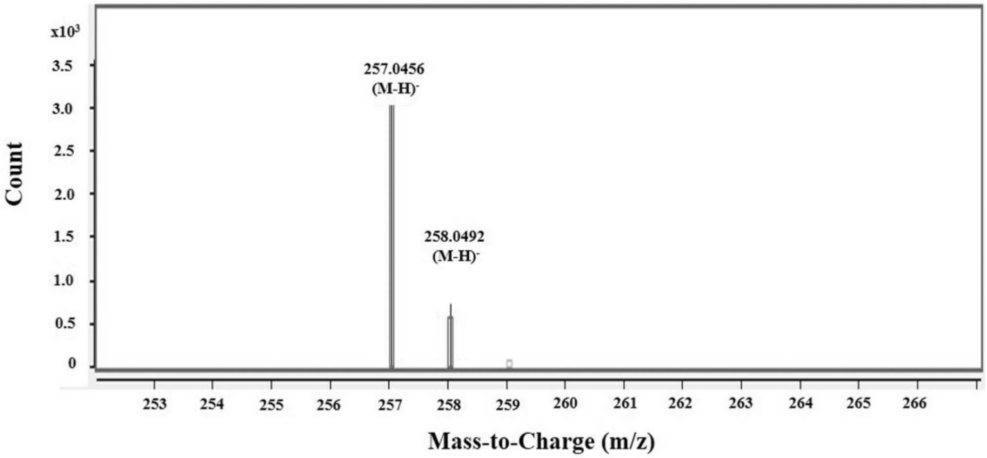
x10^3
3.5
3.0
2.5
2.0
1.5
1.0
0.5
0
Count
257.0456
(M-H)-
258.0492
(M-H)-
253 254 255 256 257 258 259 260 261 262 263 264 265 266
Mass-to-Charge (m/z)

C.

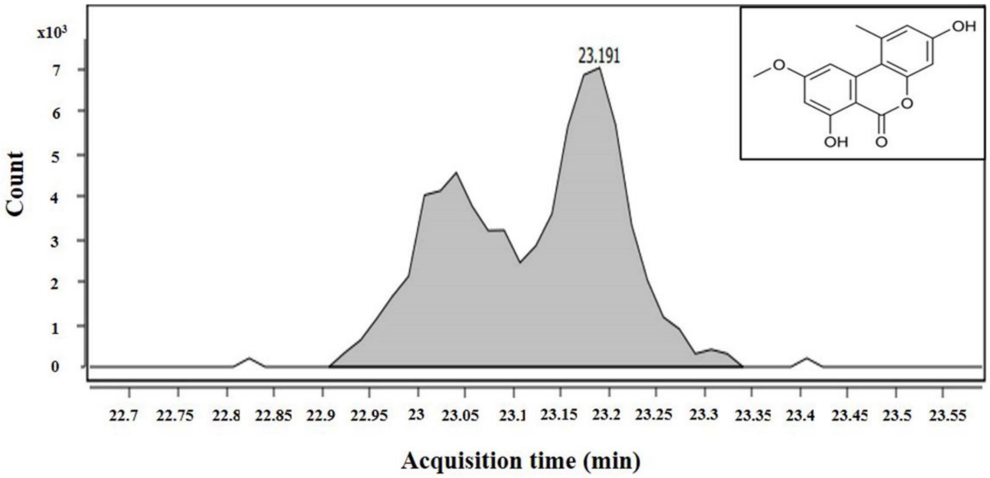
x10³
7
6
5
4
3
2
1
0
Count
23.191
OH
O
OH
O
O
22.7
22.75
22.8
22.85
22.9
22.95
23
23.05
23.1
23.15
23.2
23.25
23.3
23.35
23.4
23.45
23.5
23.55
Acquisition time (min)

D.

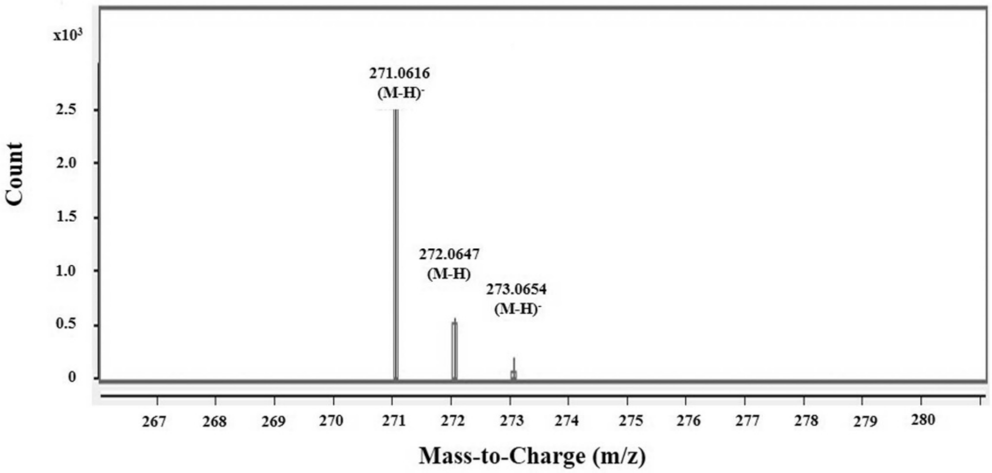
x10³
2.5
2.0
1.5
1.0
0.5
0
Count
271.0616
(M-H)-
272.0647
(M-H)
273.0654
(M-H)-
267
268
269
270
271
272
273
274
275
276
277
278
279
280
Mass-to-Charge (m/z)

E.

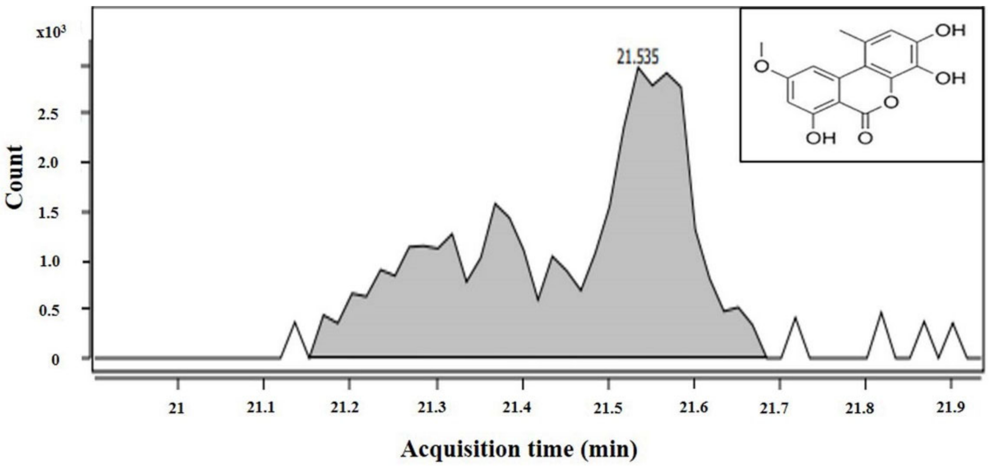
x10³
Count
2.5
2.0
1.5
1.0
0.5
0
21.535
OH
OH
OH
O
O
21
21.1
21.2
21.3
21.4
21.5
21.6
21.7
21.8
21.9
Acquisition time (min)

F.

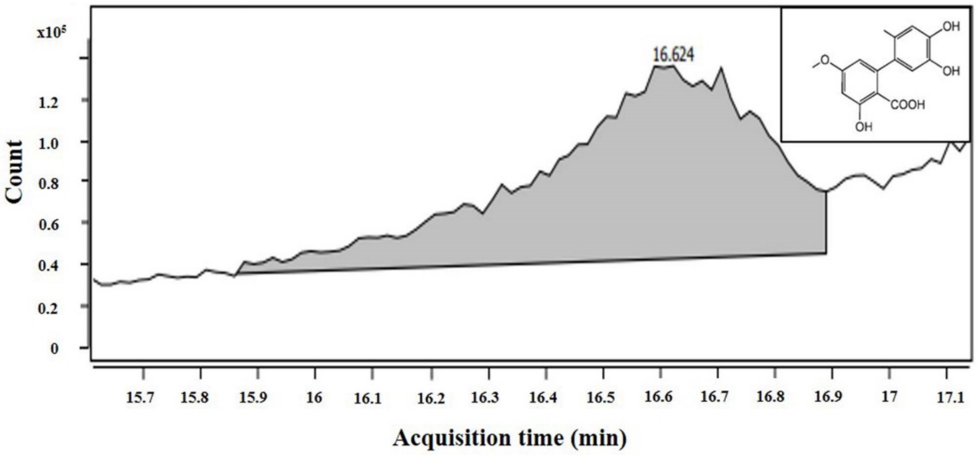
x10⁵
Count
1.2
1.0
0.8
0.6
0.4
0.2
0
16.624
OH
OH
COOH
OH
15.7
15.8
15.9
16
16.1
16.2
16.3
16.4
16.5
16.6
16.7
16.8
16.9
17
17.1
Acquisition time (min)

G.

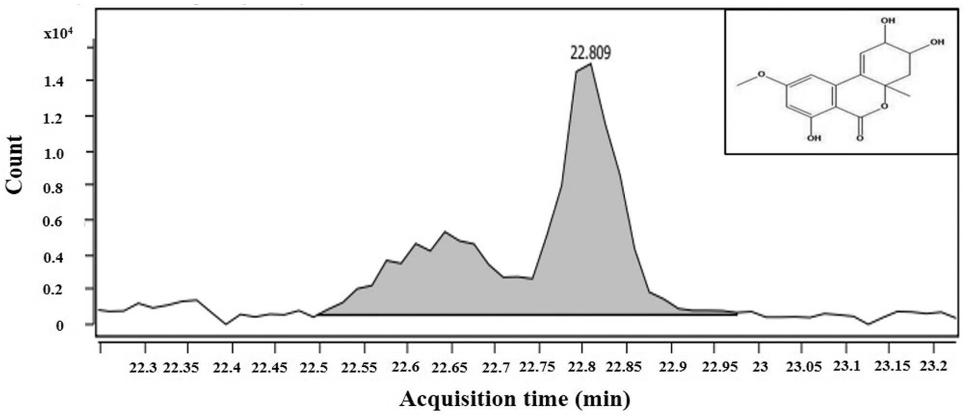

Figure S2. Extracted ion chromatograms (EIC) and mass (MS) spectra of AOH, AME, 4-OH-AME, ALN, and ALT. (A) EIC and (B) MS spectrum for AOH, (C) EIC and (D) MS spectrum for AME, (E) EIC for 4-OH-AME, (F) EIC for ALN, and (G) EIC for ALT in culture extracts of *A. alternata* KACC 411286. (Inset) AOH structure in (A), AME structure in (C), 4-OH-AME structure in (E), ALN structure in (F), and ALT structure in (G).

A.

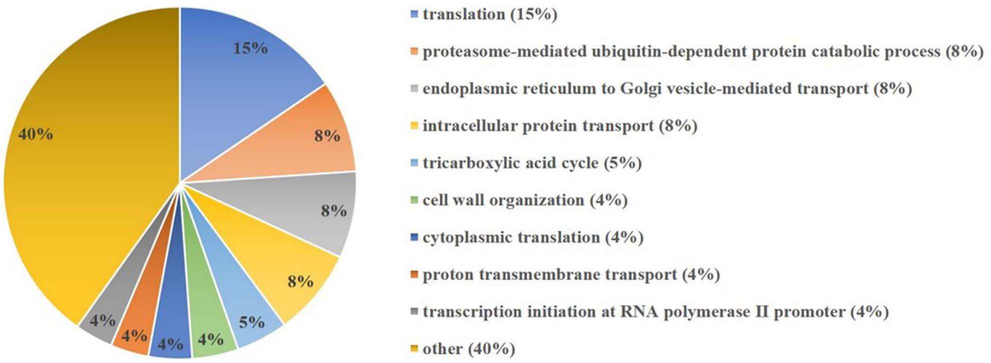

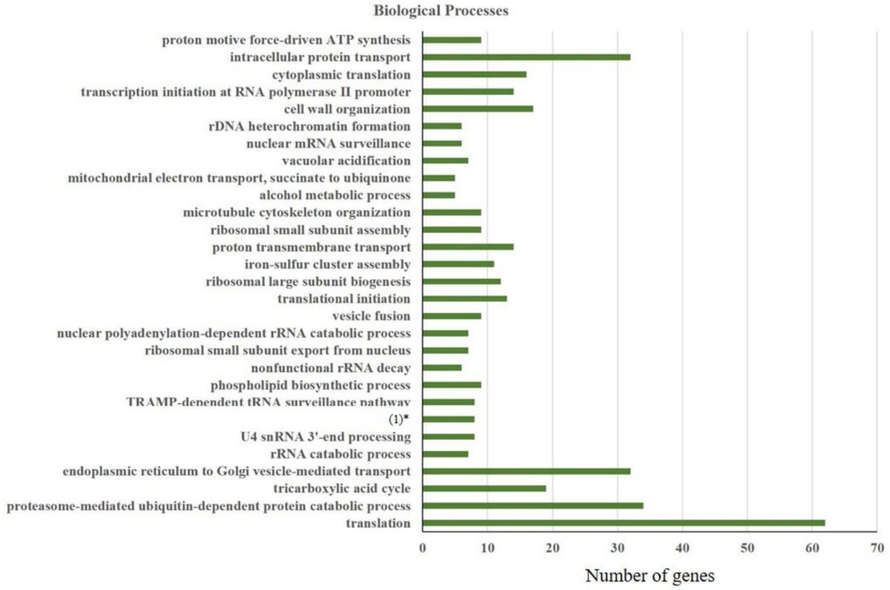

(1)* exonucleolytic trimming to generate mature 3'-end of 5.8S rRNA from tricistronic rRNA transcript (SSU-rRNA, 5.8S rRNA, LSU-rRNA)

B.

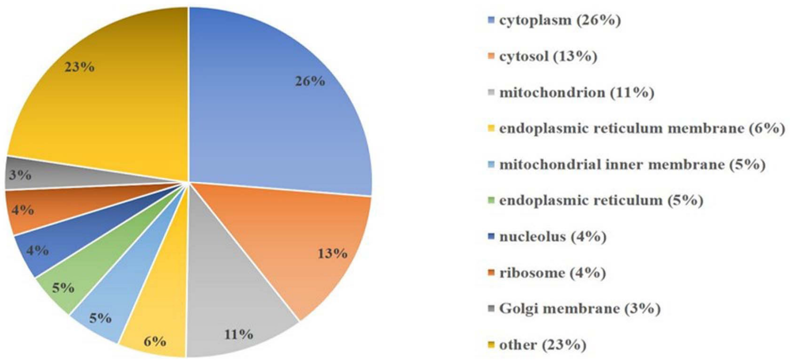
26%
13%
11%
6%
5%
5%
4%
4%
3%
23%
cytoplasm (26%)
cytosol (13%)
mitochondrion (11%)
endoplasmic reticulum membrane (6%)
mitochondrial inner membrane (5%)
endoplasmic reticulum (5%)
nucleolus (4%)
ribosome (4%)
Golgi membrane (3%)
other (23%)

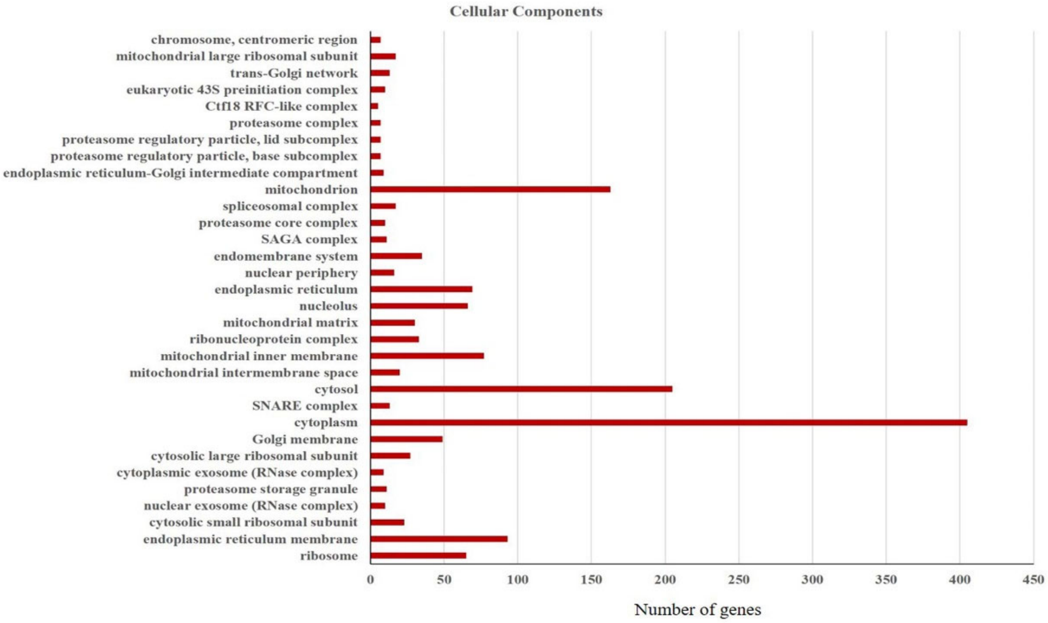
Cellular Components
chromosome, centromeric region
mitochondrial large ribosomal subunit
trans-Golgi network
eukaryotic 43S preinitiation complex
Ctf18 RFC-like complex
proteasome complex
proteasome regulatory particle, lid subcomplex
proteasome regulatory particle, base subcomplex
endoplasmic reticulum-Golgi intermediate compartment
mitochondrion
spliceosomal complex
proteasome core complex
SAGA complex
endomembrane system
nuclear periphery
endoplasmic reticulum
nucleolus
mitochondrial matrix
ribonucleoprotein complex
mitochondrial inner membrane
mitochondrial intermembrane space
cytosol
SNARE complex
cytoplasm
Golgi membrane
cytosolic large ribosomal subunit
cytoplasmic exosome (RNase complex)
proteasome storage granule
nuclear exosome (RNase complex)
cytosolic small ribosomal subunit
endoplasmic reticulum membrane
ribosome
0
50
100
150
200
250
300
350
400
450
Number of genes

C.

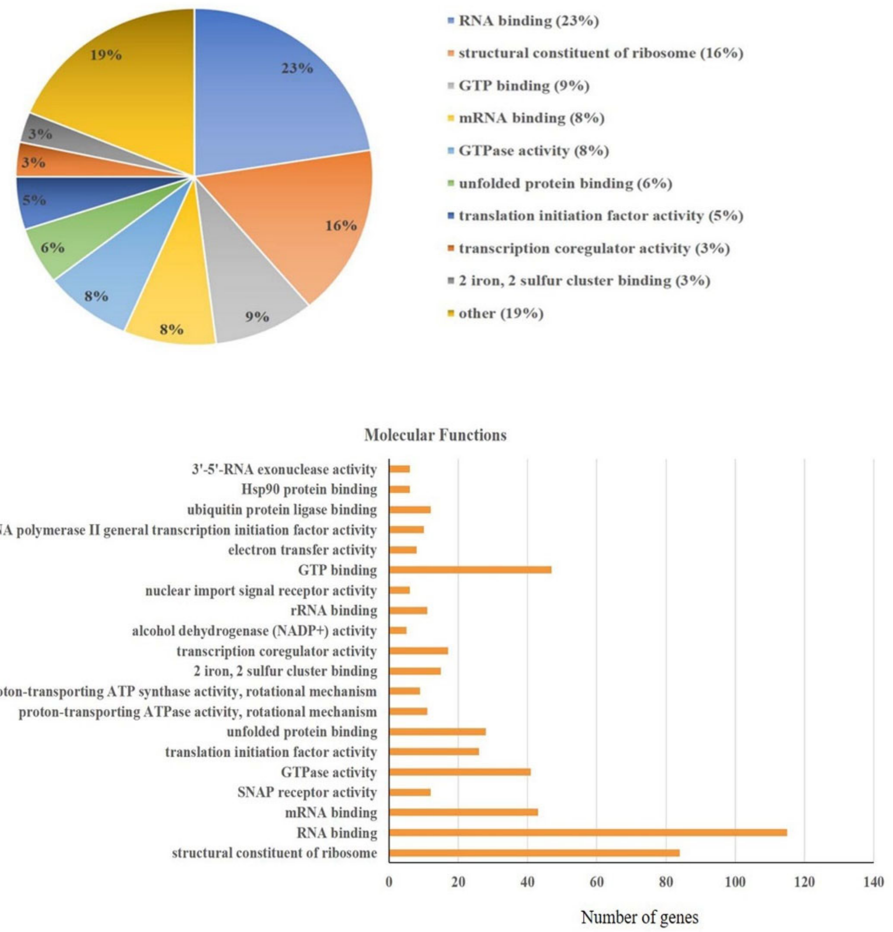

Figure S3. Gene Ontology (GO) classification of predicted proteins in *A. alternaria* KACC 411286. Assignment of GO terms for (A) biological processes, (B) cellular components, and (C) molecular functions.

A.

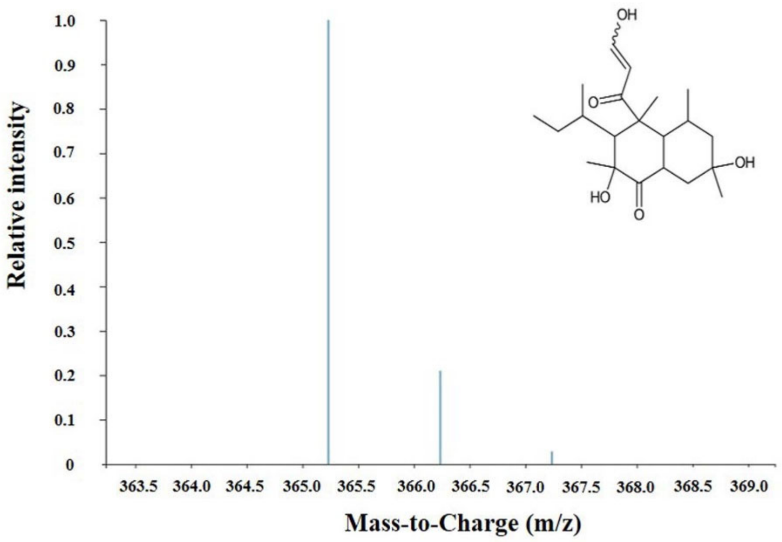

B.

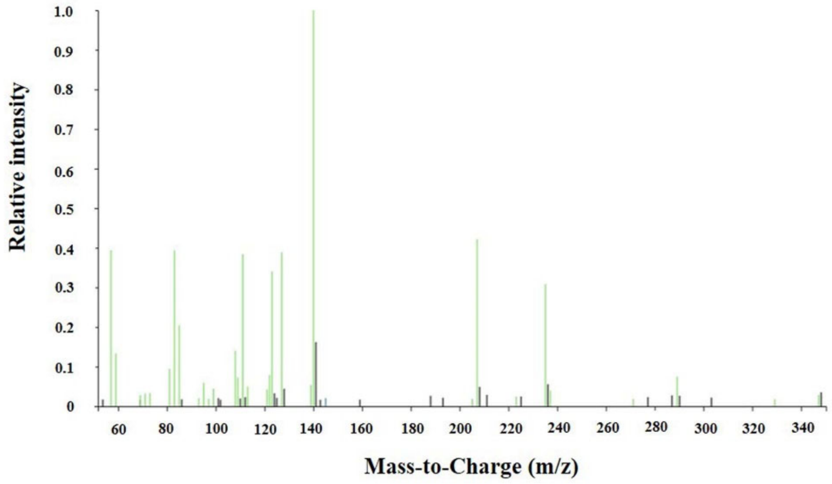

Figure S4. MS and MS/MS spectra of betaenone C identified in the culture extracts of *A. alternaria* KACC 411286 using SIRUS4. (A) MS and (B) MS/MS spectra of betaenone C. (Inset) betaenone C structure in (A)

Supplementary Tables

Table S1. Primer sequences for RT-qPCR analysis of AOH-derived toxin biosynthetic genes.

**Gene**	**Orientation**	**Primer sequence (5′ → 3′)**
moxI	Forward	ACGGCAAACAACCAGCGA
	Reverse	AACGTCCTGACCGTGCAA
sdrI	Forward	TCGCGTTGGTGCATCTGT
	Reverse	GCAGAGAAGACATCGGGCA
doxI	Forward	TCCTGCCCATGTATGTCGC
	Reverse	AAACCGGTATTGCGCCCA
β-tubulin	Forward	GCGCATGAACGTCTACTTCA
	Reverse	GGCACGAACTTGTTGTTGGA

Table S2. Lengths of ten contigs from the draft genome assembly for *A. alternata* KACC 411286.

Contig number	Length (bp)	Coverage (x)
Contig_1	7,473,212	72
Contig_2	5,512,314	73
Contig_3	3,378,571	72
Contig_4	3,049,134	72
Contig_5	2,824,231	73
Contig_6	2,774,350	73
Contig_7	2,511,040	72
Contig_8	2,438,581	72
Contig_9	2,391,645	71
Contig_10	1,851,681	70

Table S3. Genome assembly data for *A. alternata* KACC 411286.

Item	Number
Contigs	10
Total bases (bp)	34,204,759
rRNA	15
5S rRNA	9
5.8S rRNA	2
18S rRNA	2
28S rRNA	2
tRNA	122
Protein-coding gene	13,942
mRNA	13,942
Exon	36,834
CDS [1]	13,942
Functionally annotated gene	13,552

[1]CDS indicates coding sequence

Table S4. Detailed information of Gene Ontology (GO) classification of predicted proteins in *A. alternata* KACC 411286.

GO ID	Seq Num	GO term	Category
GO:0006412	62	translation	biological_process
GO:0043161	34	proteasome-mediated ubiquitin-dependent protein catabolic process	biological_process
GO:0006888	32	endoplasmic reticulum to Golgi vesicle-mediated transport	biological_process
GO:0006886	32	intracellular protein transport	biological_process
GO:0006099	19	tricarboxylic acid cycle	biological_process
GO:0071555	17	cell wall organization	biological_process
GO:0002181	16	cytoplasmic translation	biological_process
GO:1902600	14	proton transmembrane transport	biological_process
GO:0006367	14	transcription initiation at RNA polymerase II promoter	biological_process
GO:0006413	13	translational initiation	biological_process
GO:0042273	12	ribosomal large subunit biogenesis	biological_process
GO:0016226	11	iron-sulfur cluster assembly	biological_process
GO:0008654	9	phospholipid biosynthetic process	biological_process
GO:0006906	9	vesicle fusion	biological_process
GO:0000226	9	ribosomal small subunit assembly	biological_process
GO:0000028	9	microtubule cytoskeleton organization	biological_process
GO:0015986	9	proton motive force-driven ATP synthesis	biological_process
GO:0000467	8	U4 snRNA 3'-end processing	biological_process
GO:0071038	8	exonucleolytic trimming to generate mature 3'-end of 5.8S rRNA from tricistronic rRNA transcript (SSU-rRNA, 5.8S rRNA, LSU-rRNA)	biological_process
GO:0034475	8	TRAMP-dependent tRNA surveillance pathway	biological_process
GO:0016075	7	rRNA catabolic process	biological_process
GO:0071035	7	ribosomal small subunit export from nucleus	biological_process
GO:0000056	7	nuclear polyadenylation-dependent rRNA catabolic process	biological_process
GO:0007035	7	vacuolar acidification	biological_process
GO:0070651	6	nonfunctional rRNA decay	biological_process
GO:0000183	6	nuclear mRNA surveillance	biological_process
GO:0071028	6	rDNA heterochromatin formation	biological_process
GO:0006066	5	alcohol metabolic process	biological_process
GO:0006121	5	mitochondrial electron transport, succinate to ubiquinone	biological_process

Table S4 (Cont'd).

GO ID	Seq Num	GO term	Category
GO:0005840	65	ribosome	Cellular component
GO:0005789	93	endoplasmic reticulum membrane	Cellular component
GO:0022627	23	cytosolic small ribosomal subunit	Cellular component
GO:0000176	10	nuclear exosome (RNase complex)	Cellular component
GO:0034515	11	proteasome storage granule	Cellular component
GO:0000177	9	cytoplasmic exosome (RNase complex)	Cellular component
GO:0022625	27	cytosolic large ribosomal subunit	Cellular component
GO:0000139	49	Golgi membrane	Cellular component
GO:0005737	405	cytoplasm	Cellular component
GO:0031201	13	SNARE complex	Cellular component
GO:0005829	205	cytosol	Cellular component
GO:0005758	20	mitochondrial intermembrane space	Cellular component
GO:0005743	77	mitochondrial inner membrane	Cellular component
GO:1990904	33	ribonucleoprotein complex	Cellular component
GO:0005759	30	mitochondrial matrix	Cellular component
GO:0005730	66	nucleolus	Cellular component
GO:0005783	69	endoplasmic reticulum	Cellular component
GO:0034399	16	nuclear periphery	Cellular component
GO:0012505	35	endomembrane system	Cellular component
GO:0000124	11	SAGA complex	Cellular component
GO:0005839	10	proteasome core complex	Cellular component
GO:0005681	17	spliceosomal complex	Cellular component
GO:0005739	163	mitochondrion	Cellular component
GO:0005793	9	endoplasmic reticulum-Golgi intermediate compartment	Cellular component
GO:0008540	7	proteasome regulatory particle, base subcomplex	Cellular component
GO:0000502	7	proteasome regulatory particle, lid subcomplex	Cellular component
GO:0008541	7	proteasome complex	Cellular component
GO:0031390	5	Ctf18 RFC-like complex	Cellular component
GO:0016282	10	eukaryotic 43S preinitiation complex	Cellular component

Table S4 (Cont'd).

GO ID	Seq Num	GO term	Category
GO:0005802	13	trans-Golgi network	Cellular component
GO:0005762	17	mitochondrial large ribosomal subunit	Cellular component
GO:0000775	7	chromosome, centromeric region	Cellular component
GO:0003723	115	RNA binding	Molecular function
GO:0003735	84	structural constituent of ribosome	Molecular function
GO:0005525	47	GTP binding	Molecular function
GO:0003729	43	mRNA binding	Molecular function
GO:0003924	41	GTPase activity	Molecular function
GO:0051082	28	unfolded protein binding	Molecular function
GO:0003743	26	translation initiation factor activity	Molecular function
GO:0003712	17	transcription coregulator activity	Molecular function
GO:0051537	15	2 iron, 2 sulfur cluster binding	Molecular function
GO:0005484	12	SNAP receptor activity	Molecular function
GO:0031625	12	ubiquitin protein ligase binding	Molecular function
GO:0046961	11	proton-transporting ATPase activity, rotational mechanism	Molecular function
GO:0019843	11	rRNA binding	Molecular function
GO:0016251	10	RNA polymerase II general transcription initiation factor activity	Molecular function
GO:0046933	9	proton-transporting ATP synthase activity, rotational mechanism	Molecular function
GO:0009055	8	electron transfer activity	Molecular function
GO:0061608	6	nuclear import signal receptor activity	Molecular function
GO:0000175	6	Hsp90 protein binding	Molecular function
GO:0051879	6	3'-5'-RNA exonuclease activity	Molecular function
GO:0008106	5	alcohol dehydrogenase (NADP+) activity	Molecular function

Table S5. Different types of SM BGCs detected in genomes of *A. alternata* KACC 411286 and *A. alternata* Z7.

Fungal strain	NRPS	NRPS-like	PKS	PKS-like	TC	TPS	DMAT	Hybrid	RiPP	NAPAA	Total
A. alternata KACC 411286	3	5	8	0	14	2	0	6	1	1	40
A. alternata Z7	6	7	10	0	7	0	0	0	1	0	31

NRPS, PKS, TC, TPS, DMAT, RiPP, and NAPAA indicate non-ribosomal peptide synthetase, polyketide synthase, terpene cyclase, terpene precursor synthase, dimethylallyl tryptophan synthase, and ribosomally synthesized and post-translationally modified peptide, and non-alpha poly-amino acid, respectively.

Table S6. Identification of SMs produced by *A. alternata* KACC 411286 using LC-MS/MS database at GNPS site or SIRIUS4.

Compound name	Molecular formula	Tool for identification of SMs
Altenusin	$C_{15}H_{14}O_6$	GNPS, SIRIUS4
6-Gingerol	$C_{17}H_{26}O_4$	GNPS, SIRIUS4
Anisomysin	$C_{14}H_{19}NO_4$	GNPS
Narcotine	$C_{22}H_{23}NO_7$	GNPS
Dioctyl sulfosuccinate	$C_{20}H_{37}NaO_7S$	GNPS
(1E,4E)-1,5-bis(4-methoxyphenyl)penta-1,4-dien-3-one	$C_{19}H_{18}O_3$	GNPS
Methyl 3-(3,4-dihydroxyphenyl)-2-[[4-hydroxy-3-(3-methylbut-2-enyl)phenyl]methyl]-4-methoxy-5-oxofuran-2-carboxylate	$C_{30}H_{34}O_8$	GNPS
Alternariol 5-O-methyl ether-4′-O-sulfate	$C_{15}H_{12}O_8S$	SIRIUS4
Betaenone C	$C_{21}H_{34}O_5$	SIRIUS4

Table S7. Functions of six ALT biosynthetic genes and comparison of the size of each gene and the number of introns in the gene from *A. alternata* KACC 411286 with those from *A. alternata* ATCC 66981.

Gene	Predicted function [1]	Gene size (bp) [2,3]	Number of intron [2,3]	Protein size [2,3] (Number of amino acid)	Transcriptional orientation of gene
doxI	Extradiol dioxygenase	1,102 (1,102)	2 (2)	325 (325)	-
sdrI	Shor chain dehydrogenase	872 (749)	1 (1)	271 (230)	+
aohR	Gal4-like Zn(II)$_2$Cys$_6$ transcription factor	1,662 (1,662)	0 (0)	553 (553)	-
moxI	FAD-dependent monooxygenase	1,480 (1,318)	1 (1)	475 (421)	-
omtI	O-methyltransferase	1,515 (1,428)	4 (5)	417 (379)	-
pksI	Polyketide synthase	5,796 (5,000)	3 (3)	1,763 (1,484)	+

[1]The predicted functions of ALT biosynthetic genes were obtained from the previous study [11].

[2]The sizes of each gene and its corresponding protein, and the number of intron were generated in this study for *A. alternata* KACC 411286 and were obtained from the publicly available information at the NCBI website for *A. alternata* ATCC 66981.

[3]The number in the parenthesis indicates that from *A. alternata* ATCC 66981.
